# Challenges Predicting Ligand-Receptor Interactions of Promiscuous Proteins: The Nuclear Receptor PXR

**DOI:** 10.1371/journal.pcbi.1000594

**Published:** 2009-12-11

**Authors:** Sean Ekins, Sandhya Kortagere, Manisha Iyer, Erica J. Reschly, Markus A. Lill, Matthew R. Redinbo, Matthew D. Krasowski

**Affiliations:** 1 Collaborations in Chemistry, Jenkintown, Pennsylvania, United States of America; 2 Department of Pharmaceutical Sciences, University of Maryland, Baltimore, Maryland, United States of America; 3 Department of Pharmacology, University of Medicine & Dentistry of New Jersey (UMDNJ)-Robert Wood Johnson Medical School, Piscataway, New Jersey, United States of America; 4 Department of Microbiology and Immunology, Drexel University College of Medicine, Philadelphia, Pennsylvania, United States of America; 5 Department of Pathology, University of Pittsburgh, Pittsburgh, Pennsylvania, United States of America; 6 Department of Medicinal Chemistry and Molecular Pharmacology, Purdue University, West Lafaytte, Indiana, United States of America; 7 Department of Chemistry, University of North Carolina at Chapel Hill, Chapel Hill, North Carolina, United States of America; 8 Department of Biochemistry and Biophysics, University of North Carolina at Chapel Hill, Chapel Hill, North Carolina, United States of America; 9 The Lineberger Comprehensive Cancer Center, University of North Carolina at Chapel Hill, Chapel Hill, North Carolina, United States of America; University of Houston, United States of America

## Abstract

Transcriptional regulation of some genes involved in xenobiotic detoxification and apoptosis is performed via the human pregnane X receptor (PXR) which in turn is activated by structurally diverse agonists including steroid hormones. Activation of PXR has the potential to initiate adverse effects, altering drug pharmacokinetics or perturbing physiological processes. Reliable computational prediction of PXR agonists would be valuable for pharmaceutical and toxicological research. There has been limited success with structure-based modeling approaches to predict human PXR activators. Slightly better success has been achieved with ligand-based modeling methods including quantitative structure-activity relationship (QSAR) analysis, pharmacophore modeling and machine learning. In this study, we present a comprehensive analysis focused on prediction of 115 steroids for ligand binding activity towards human PXR. Six crystal structures were used as templates for docking and ligand-based modeling approaches (two-, three-, four- and five-dimensional analyses). The best success at external prediction was achieved with 5D-QSAR. Bayesian models with FCFP_6 descriptors were validated after leaving a large percentage of the dataset out and using an external test set. Docking of ligands to the PXR structure co-crystallized with hyperforin had the best statistics for this method. Sulfated steroids (which are activators) were consistently predicted as non-activators while, poorly predicted steroids were docked in a reverse mode compared to 5α-androstan-3β-ol. Modeling of human PXR represents a complex challenge by virtue of the large, flexible ligand-binding cavity. This study emphasizes this aspect, illustrating modest success using the largest quantitative data set to date and multiple modeling approaches.

## Author Summary

Promiscuous proteins generally bind a large array of diverse ligand structures. This may be facilitated by a very large binding site, multiple binding sites, or a flexible binding site that can adjust to the size of the ligand. These aspects also increase the complexity of predicting whether a molecule will bind or not to such proteins which frequently function as exogenous compound sensors to respond to toxic stress. For example, transporters may prevent absorption of some molecules, and enzymes may convert them to more readily excretable compounds (or alternatively activate them prior to further clearance by other detoxification enzymes). Nuclear hormone receptors may respond to ligands and then affect downstream gene expression to upregulate both enzymes and transporters to increase the clearance for the same or different molecules. We have assessed the ability of many different ligand-based and structure-based computational approaches to model and predict the activation of human PXR by steroidal compounds. We find the most effective computational approach to identify potential steroidal PXR agonists which are clinically relevant due to their widespread use in clinical medicine and the presence of mimics in the environment.

## Introduction

Promiscuous proteins generally bind a large array of diverse ligand structures. These proteins include enzymes like cytochrome P450s (e.g. CYP3A4, EC 14.13.97), transporters such as P-glycoprotein (ABCB1), the human ether-a-go-go related gene (hERG, K_v_11.1) potassium channel and nuclear hormone receptors (NHRs) such as the pregnane X receptor (PXR; NR1I2; also known as SXR or PAR) [1]. This promiscuous binding may be facilitated by a very large binding site, multiple (overlapping) binding sites, or a flexible binding site that can adjust to the size of the ligand. Intrinsic disorder in the protein may also have a role [2],[3]. These proteins described above are also particularly important as xenobiotic sensors and represent key mechanisms to respond to toxic stress.

The human PXR [4]–[6] transcriptionally regulates genes involved in xenobiotic metabolism and excretion, as well as other cellular processes such as apoptosis [7]–[11]. Human PXR has a very broad specificity for ligands as exemplified by the structurally diverse array of activators including endogenous (bile acids, steroid hormones, fat-soluble vitamins) and exogenous (prescription and herbal drugs, and environmental chemicals) compounds. Activation of human PXR can cause drug-drug interactions [4],[5] or result in physiological effects ranging from ameliorating cholestatic injury to the liver, altering bone homeostasis, and causing cell proliferation [12]. As PXR represents a potential target for pharmacologic modulation in disease, it is therefore becoming even more important to develop methods that can identify whether a molecule is likely to be a PXR agonist [13]. Currently there are five high-resolution crystal structures of human PXR [14]–[18] available in the Protein Data Bank (PDB) (and another structure to be deposited [19]). The structures have provided atomic level details that have led to a greater understanding of the ligand binding domain (LBD) and the structural features involved in ligand-receptor interactions [9], [10], [12]–[15]. The co-crystallized ligands include the natural products hyperforin (active component of the herbal anti-depressant St. John's wort) and colupulone (from hops), the steroid 17β-estradiol, the synthetic compounds SR12813, T1317 and the antibiotic rifampicin. These ligands span a range of molecular sizes (M.Wt range 272.38 – 713.81Da, mean 487.58±147.25Da, ) and are predicted as generally hydrophobic (calculated ALogP [20] 3.54–10.11, mean 5.54±2.41). The cavernous ligand binding pocket (LBP) with a volume >1350 Å^3^ accepts molecules of these widely varying dimensions and chemical properties, and is likely capable of binding small molecules in multiple orientations [21]. This complicates overall prediction of whether a small molecule is likely to be classified as a PXR agonist using traditional structure-based virtual screening methods like docking [13],[22]. With regard to this, we have previously shown that the widely used structure-based docking methods FlexX and GOLD performed relatively poorly in predicting human PXR agonists [7],[16] and this is perhaps not surprising based on the observations described above.

An alternative method, which has been found to be valuable elsewhere in drug discovery, particularly when there may not be an available crystal structure of the target protein, uses a ligand-based approach. In this case a series of small molecule structures with PXR agonist activity data can be used to facilitate a structure activity relationship (SAR). When the biological activity data is continuous this will enable a quantitative structure activity relationship (QSAR) [23]–[25]. One widely used computational technology produces pharmacophores [20]–[23], which represent models that encode the key chemical features important for biological activity. Human PXR agonist pharmacophore models have been shown to possess hydrophobic, hydrogen bond acceptor and hydrogen bond donor features, consistent with the crystallographic structures of human PXR ligand-receptor complexes [26]–[29]. These pharmacophore models have predominantly used structurally diverse ligands in the training set and have the limitation in most cases of compiling data from multiple laboratories using different experimental protocols, ultimately forcing binary classifications of ligands for the training sets (i.e., activating versus non-activating). Most of the models so far use EC_50_ data, a measure of receptor transactivation. Although binding assays have been done with human PXR, they are problematic given the low affinity of most PXR activators. As a result, there is little radioligand binding data in the literature other than competition experiments with radiolabeled SR12813.

To date there have been few attempts to build ligand-based models around a large structurally narrow set of PXR activators. The absence of large sets of quantitative data for PXR agonists has restricted QSAR models to a relatively small universe of molecules compared to the known drugs, drug-like molecules, endobiotics and xenobiotics in general [30]. The PXR data limitation has resulted in the use of various machine learning methods (e.g support vector machine, recursive partitioning etc.) when the biological data is binary in nature (e.g. activating or binding versus non-activating / non-binding) [13],[22],[25],[30].

As part of an ongoing analysis of NHRs [25]–[28], we have generated a large cadre of experimental data for classes of steroidal compounds, namely androstanes, estratrienes, pregnanes and bile acids/salts [31]. The advantages of using steroidal compounds for QSAR are that they are amenable to common alignments based on the steroidal backbone. For, example steroids represented the first datasets used for comparative molecular fields analysis (CoMFA) [32] and have been widely used as a benchmark for other methods such as comparative molecular similarity analysis (CoMSIA)[33]. Pharmacophore methods, in contrast, generally do not require the rigid alignment methods and have found use with more diverse structure sets [28],[31]. Using this large quantitative data set of PXR activators, we applied various ligand-based computational methods including Bayesian modeling with 2D fingerprints. We also compared the results from QSAR approaches to molecular docking into the six available human PXR crystal structures.

Modeling of a broad specificity receptor such as PXR represents a challenge for *in silico* modeling and it is invaluable to know what approaches prove successful, if any. Ideally, these methods will also translate to modeling approaches for other broad specificity enzymes, transporters and ion channels [1], or other promiscuous proteins [34]. We are not aware of any similar studies using a comparative approach to predicting ligand-protein interactions for promiscuous proteins. This study also provides further insights into PXR-steroid interactions which have not been well studied [19] and is clinically relevant due to the widespread use of steroidal compounds and steroid mimics (e.g. oral contraceptives [35], for inflammation and as cancer treatments etc.) in clinical medicine [36], as well as the increasing problem of environmental contamination by endocrine disruptors [24].

## Results

### Docking

All compounds shown in Table S2 were docked to the six human PXR crystal structures using GOLD which we have used previously for docking diverse compounds into the human PXR structure [22]. All six crystal structures superimposed with a backbone root mean squared deviation of 0.5 Å suggesting that they had very similar structures and their co-crystallized ligands bound to the same binding pocket (Figure S1). The docking scores for all the compounds (Table S2) were in the range of 36 to 77 for all the crystal structures and their corresponding Tanimoto similarity scores to 5α-androstan-3β-ol and the crystal ligand 17β-estradiol using MDL public keys were between 0.4 and 1.

To evaluate docking results, we compared docking scores for classifying compounds as activators or non-activators of PXR. Using an EC_50_ value of 10 µM as a cutoff the compounds listed in Table S2 were classified as activators (30 compounds) and non-activators (89 compounds). These results were compared to the classification obtained from the docking studies. The overall accuracy (Q values) were in the range of 35 to 55 % for models that used 5α-androstan-3β-ol based similarity scores as weights to the goldscore, while the Q values were in the range of 47 to 58% for models that were generated with goldscores weighted with 17β-estradiol based similarity scores (Table 1). The Matthews coefficient C showed a modest prediction rate with the best score for docking of compounds to PXR crystal structure 1M13. Further changing the cutoff values to either 100 µM or 40 µM did not improve the prediction rates. The Q value for a model computed by averaging all the models with 5α-androstan-3β-ol weighted goldscore was 46% and for the average model with 17β-estradiol the weighted goldscore was 51%.

**Table 1:** Docking results for all of the 119 molecules to the six hPXR crystal structures and the combined model in terms of statistical parameters namely sensitivity (SE), specificity (SP), overall prediction accuracy (Q) and matthews correlation coefficient (C) are listed for models predicted with 17β-estradiol similarity weighted goldscores and the values in parenthesis are for models predicted with 5α-androstan-3β-ol similarity weighted goldscores.

Structure	SE (%)	SP (%)	Q (%)	C (%)
1M13 (hyperforin )	66.67(56.7)	55.06(53.9)	57.98(54.6)	0.19(0.09)
1NRL (SR12813)	46.67(60)	47.19(41.6)	47.06(46.2)	−0.05(0.01)
1SKX (rifampicin)	53.33(70)	50.56(39.3)	51.26(47.1)	0.03(0.08)
2O9I (T0901317)	53.33(40)	50.56(40.4)	51.26(40.3)	0.03(−0.17)
2QNV (colupulone)	53.33(46.7)	52.81(31.5)	52.94(35.3)	0.05(−0.19)
EST (estradiol)	53.33(56.67)	50.56(49.44)	51.26(51.26)	0.02(0.05)
AVG	52 (55)	50.34 (42.69)	50.76(45.79)	0.02(−0.02)

The values in AVG represent the average prediction rates.

**Figure 1:**
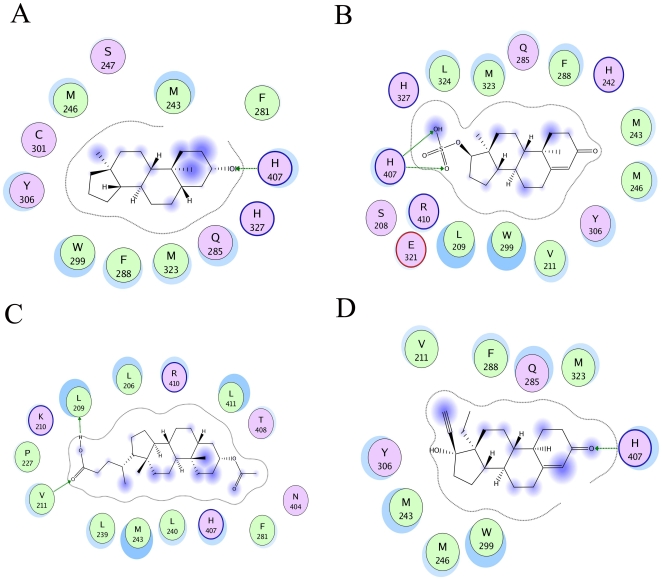
Schematic representation of the binding mode of A. 5α-Androstan-3β-ol B epitestosterone sulfate C lithocholic acid acetate and D levonorgestrol in the binding site of crystal structure of human PXR protein (PDB code: 1M13). The binding site residues are colored by their nature, with hydrophobic residues in green and charged residues in purple. Blue spheres and contours indicate matching regions between ligand and receptors. The schematic representations were generated using the LIGX option in MOE.

Although the overall performance of docking produced rather modest results for classification the results for individual classes of compounds was better than average. In the best classification model (compounds docked to crystal structure 1M13 and weighted with 17β-estradiol based similarity scores), 20 out of 30 PXR activators and 49 out of 89 non-activators were predicted correctly. Among the androstanes, 6 out of 11 compounds were predicted correctly as activators and 9 out of 14 compounds were classified as non-activators (Table S2). Among the bile salts, all 4 activators and 22 out of 46 non-activators were predicted correctly. Among the estratrienes, 5 out of 7 activators were predicted correctly, while the 4 non-activators were predicted as activators (Table S2). The reason for this mis-classification was due to the high similarity scores of the estrogens with 17β-estradiol. In the pregnane class, 4 out of 7 activators and 16 out of 20 non-activators were correctly classified (Table S2). Some examples of molecules in their binding modes with PXR structure 1M13 are shown in Figure 1.

### 2D-Classification: Bayesian Models

All 115 compounds shown in Table S2 were used to generate a Bayesian classification model [37], using a definition of active as a compound having an EC_50_ for PXR activation of less than 10 µM. Using molecular function class fingerprints of maximum diameter 6 (FCFP_6) and 8 interpretable descriptors (AlogP, molecular weight, rotatable bonds, number of rings, number of aromatic rings, hydrogen bond acceptor, hydrogen bond donor and polar surface area) a model was developed with a receiver operator curve (ROC) statistic for leave one out cross validation of 0.84. In addition to the leave one out cross validation, further validation methods were undertaken. After leaving 20% of the compounds out 100 times the ROC is 0.84±0.08, concordance 73.2 %±8.94, specificity 69.14%±12.12, and sensitivity 84.11%±18.04. The Bayesian method appears to have good model statistics for internal cross validation of steroids. These statistics suggest the model is stable and not over-trained as the ROC values are essentially identical to that obtained with leave one out cross validation.

We have additionally used this model to classify a previously used diverse molecule test set [13],[22]. After removing the steroids from the test set, the Bayesian PXR model was used to rank 123 molecules (65 activators and 58 non activators). Out of the top 30 molecules scored and ranked with this model 20 (75%) were classified as activators (EC_50_ <100 µM) (Table S3). Even though the cutoff for activity for the Bayesian model is more stringent it still appears to be able to predominantly pick out the key molecular features that contribute to activity in non-steroidal compounds.

**Figure 2:**
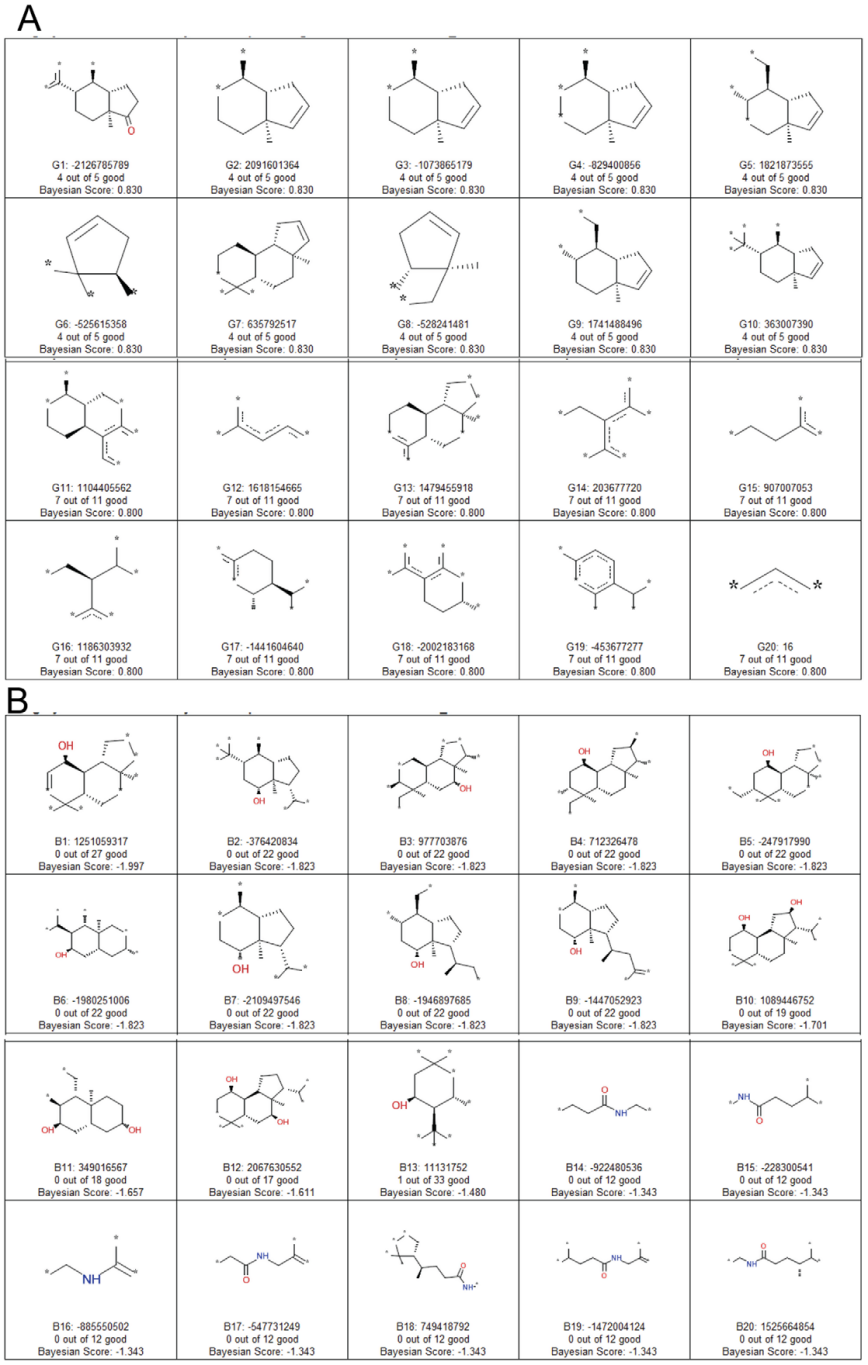
Good and bad molecular features identified in the Bayesian model using FCFP_6 fingerprints. A. Good features from FCFP_6 Bayesian model, B. Bad features from FCFP_6 Bayesian model. Asterisks can represent any atom. Numbers represent how many molecules out of the total number possessing the fingerprint are active (good) or inactive (bad).

The Bayesian model with FCFP_6 descriptors also enabled the visualization of substructure fingerprints (Figure 2) that either contributed positively or negatively to the activity classification. It appears that all positive contributing substructures are essentially hydrophobic, while negatively contributing features possess hydroxyl or other substitutions which are likely not optimally placed to facilitate interactions with hydrogen bonding features in PXR. Therefore possession of these hydrogen bond acceptor and donor features indicated in the steroidal substructures appears to be related to loss of PXR activation. The method does not readily identify where these groups should be added in contrast to methods like docking [13],[38].

### 3D-QSAR and 4D-QSAR

A major challenge in CoMFA and CoMSIA modeling is alignment of molecules, which must be defined by the user. As described in Text S1, multiple alignment approaches were attempted. Despite the use of multiple alignments, the best CoMFA and CoMSIA models consistently showed a large difference between the correlation R^2^ and cross-validated (XV-R^2^), whether modeling the entire set of steroidal compounds or the various subsets (androstanes, bile salts, pregnanes). This suggests that the CoMFA and CoMSIA models do not generalize beyond the molecules in the training set, even for a subset of steroidal compounds (Text S1, Tables S4, S5, S6, S7 and Figures S2, S3, S4, S5, S6, S7).

Using the pharmacophore approach for the individual steroids, the training set r values were quite low but increased upon inclusion of excluded volumes with variable weight and tolerances (0.81–0.93) (Table S8). All PXR pharmacophores (Figure S8) had at least 2 hydrophobes and a hydrogen bond acceptor in common (Text S1). Using the pharmacophores derived from training sets based on subsets of steroidal compounds (e.g., androstanes only) to predict the other respective subsets did not result in reliable correlations (data not shown), suggesting that highly specific pharmacophores were generated or this may be due to the addition of the excluded volumes which limits the chemical space of molecules mapping to the features. These class-specific pharmacophores may therefore only be useful for making predictions of very closely related molecules and even crossing steroidal classes may be extrapolating too far beyond the training sets.

4D-QSAR performed somewhat better than CoMSIA and CoMFA in modeling the compounds in the training sets using three atom alignments (Table S9). One potential advantage of 4D-QSAR relative to standard 3D-QSAR methods is the ability to consider an ensemble of different ligand conformations, theoretically increasing the chances of defining the active conformation. The best 4D-QSAR models are found in Table S10 and Figure S9, and generally predict steric/non-polar interactions between ligand and receptor. Although the XV-R^2^ for the best 4D-QSAR models are better than for CoMFA and CoMSIA models of the same training sets, the 4D-QSAR were poorly predictive of the activity of compounds in the test set (Table S10).

### 5D - QSAR

4D- and 5D-QSAR have the advantage of being able to select the bioactive conformation from a pool of possible binding modes in parallel to the QSAR modeling stage. We have tested three different alignment protocols in conjunction with the 5D-QSAR technique Raptor.

1. The top-1, top-2, top-5, top-10 and top-20 docking poses for each ligand from our docking studies on 2QNV were superimposed producing an alignment containing 115 – 2300 conformations for the 115 compounds in our dataset.
2. All 115 compounds were automatically aligned onto 17β-estradiol extracted from the complex structure with PXR. Already aligned compounds were automatically added as templates for aligning subsequent compounds. The order of compounds in the alignment was determined based on their experimental affinity towards PXR.
3. As in (2) but each of the four substrate classes was aligned separately on a template of the class selected by the lowest binding affinity. The individual templates are first aligned onto 17β-estradiol. The four individual alignments are then combined into a 4D set for all 115 compounds.

In the alignment protocols (2) and (3) the protein crystal structure was used as a forbidden (excluded) region. A penalty was added to the similarity score for alignment solutions that overlapped with the protein, thus physically impossible solutions were removed from the alignment. As significant protein flexibility is observed on the side chain level, all crystal structures were aligned using PyMol [39]. Side chains that have different rotamer states for different co-crystallized ligands were removed from the forbidden region definition.

Our multidimensional QSAR study (software *Raptor* [40]) was based on the same set of 115 molecules as described in the CoMFA and CoMSIA studies. The dataset was split into 95 training set compounds, and 20 test set compounds identical to the separation used in the CoMFA and CoMSIA studies. For 33 compounds only an upper limit for their K_i_ values has been experimentally determined. These molecules defined the “threshold class” (26 training, 7 test). A threshold value of 100 µM was chosen considering that the lowest affinities were measured for this dataset at approximately this value. To allow for topological and physicochemical variation at the true biological receptor with different ligands bound, the *Raptor* results were averaged over 10 individual models defining a surrogate conformational family.

For alignment (1) we were not able to derive QSAR models with predictive models for leave-5-groups-out (r^2^_CV-5_) or cross-validation values (i.e.>0.3). This is not surprising, as the identification of bioactive binding modes using docking is difficult for this system (see docking results). If we use an alignment with only the top-1 or top-2 solutions, we most probably end up with an alignment containing incorrect binding modes. Using the top-10 or top-20 binding modes generates too large a variety of contacts between ligand and binding site model that the QSAR algorithm is not able to extract the critical interactions throughout the binding site modeling phase.

**Figure 3:**
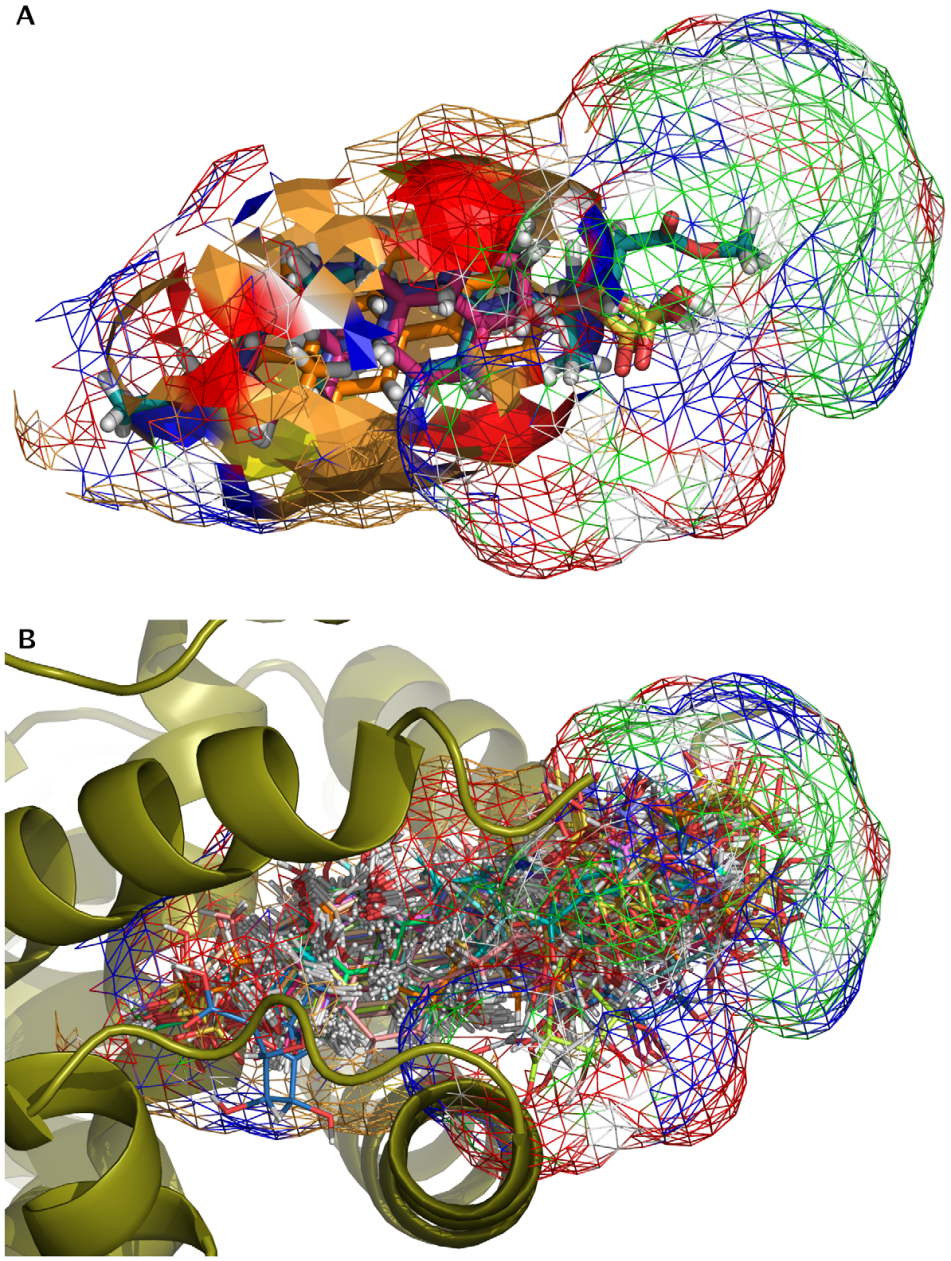
Receptor model for PXR obtained using Raptor (beige-brown, hydrophobic properties; red, hydrogen bond acceptor; blue, hydrogen-bond donor; and green, hydrogen bond donor/acceptor). The most active ligand of each of the four substrate classes aligned to each other is displayed as sticks. A: Inner shell is displayed in surface representation, outer shell in wireframe. B: The bulky right portion of the outer shell corresponds to the solvent exposed region of the ligand alignment. It is dominated by a mixed hydrogen bond donor/acceptor character in agreement with solvent exposure.

For alignment (2) a QSAR model with a r^2^_CV-5_ value of 0.55 could be generated, but with no observed correlation for the test set. For alignment (3) a QSAR model with an r^2^_CV-5_ of 0.56 was derived with a predictive r^2^ for the test set of 0.45. The superior model based on alignment (3) was due to the focused class-based alignment process (Figure 3). The maximum deviation of predicted from experimentally measured EC_50_ is 5.6 and 3.0 fold for training and test set, respectively. Significantly higher regression coefficients can hardly be expected for this dataset considering the fact that the threshold compounds have to be removed from the calculation of the regression coefficients yielding a rather small range in EC_50_ of 2.2 log units (Figure S10, Table S11). This is in contrast to the CoMFA and CoMSIA simulations where the threshold compounds have been assigned an EC_50_ value of 10,000 µM yielding a range of 4.1 log units. All except one of the 33 threshold compounds have been predicted with an EC_50_ value lower than the given threshold or maximally a factor of 6.6 fold higher. Only 5α-Androstane was predicted to have a 46 fold higher value than the threshold. Thus, the model was able to predict the affinity of compounds accurately and at the same time was able to classify weak- or non-binding molecules correctly.

## Discussion

It has been suggested that PXR forms a heterotetramer and exhibits a range of motions which are key for its functioning and preparing for coactivator binding at the Activator Function (AF-2) site [41]. The large and promiscuous ligand binding pocket of PXR accepts molecules of widely varying sizes (Table S1), and is likely capable of binding small molecules in multiple orientations. Furthermore, movement of regions of this pocket may be translated elsewhere in the protein to influence protein-protein interactions. Thus, the identification of the bioactive conformation of a ligand binding to PXR (and the effect it might have as an agonist, antagonist or allosteric antagonist [10]) and development of a ligand alignment based on these conformations represents a challenge for any computational technique. A realistic ligand alignment, however, is the basis for a reliable 3D-QSAR model. Computational methods including QSAR (3D, 4D and 5D), pharmacophores and machine learning classification models for PXR can assist in rapid prediction of whether a compound is likely to be an agonist (activator), however each method has its limitations and advantages (Table 2). For example a previous study used human PXR activation data for 30 steroidal compounds (including 9 bile acids) to create a pharmacophore with four hydrophobic features and one hydrogen bond acceptor [27]. This pharmacophore contained 5α-androstan-3β-ol (EC_50_ 0.8 µM) which contains one hydrogen bond acceptor, indicating that in contrast to the crystal structure of 17β-estradiol (published EC_50_ 20 µM) bound to human PXR with two hydrogen bonding interactions [19], hydrophobic interactions may therefore be more important for increased affinity [27]. This and other pharmacophores have been used to predict PXR interactions for antibiotics [35] which were verified *in vitro*, suggesting one use for computational approaches in combination with experimental methods.

**Table 2:** Summary of the different methods used in this study.

Method	Advantages	Limitations
Bayesian Classification with 2D Fingerprints and interpretable descriptors	Computationally fast and cheap model generation, illustration of features important for activity	Cannot deal with stereoisomers, not quantitative, requires quite large training sets
3D-QSAR :Catalyst	Can use structurally diverse molecules, can add excluded volumes, quantitative, interpretable, starts from multiple conformations.	Models may not be useful beyond a narrow compound class, conformations may not be biologically relevant
3D-QSAR: CoMFA, COMSIA	Widely used methods and useful for drug design and analog modification.	Molecules require manual alignment and this may be a major limitation in this study
4D-QSAR	Considers an ensemble of different ligand conformations to define the active conformation	Computationally expensive, Alignment strategy may be a limitation
5D-QSAR	Considers an ensemble of different ligand conformations to define the active conformation in parallel, less rigid alignment, better treatment of weak binders.	Computationally expensive, Alignment strategy may be a limitation. For alignment crystal structures may not amply take into account the protein flexibility however in Raptor this is treated explicitly, exclusion areas could be too harsh.
GOLD docking and scoring	May provide potential binding orientation with respect to pocket which could be verified by site directed mutagenesis	Relatively slow, defining the binding site is key in such a large pocket

To our knowledge there has been no comparative analysis of the steroidal classes with respect to their use as PXR agonists. The use of the Bayesian classification with 2D fingerprints represents a low computational cost approach [42] which has been used frequently with large molecule datasets [43]–[46]. Using 2D-molecular fingerprint descriptors identified regions in the training set molecules that were predominantly hydrophobic and that were important for PXR activation. Substructures with free hydroxyls as hydrogen bonding features were associated with compounds that were not activators. This is in general agreement with other studies which have used docking to try to help design out PXR activation [38]. This model was able to successfully rank a large test set (Table S3) of non-steroidal molecules, indicative that the molecular descriptors adequately captured the global properties of PXR agonists and suggests some utility.

The current study suggests that while it is generally possible to create 3D-QSAR (CoMFA, CoMSIA, Catalyst) and 4D-QSAR models that can be cross-validated, these models perform poorly when used to predict external molecules. Only the 5D-QSAR model generated displays some success in predicting external test set steroidal compounds. Three main differences between the 5D-QSAR and the 3D-QSAR studies that might contribute to the difference in performance are the less rigid alignment using Symposar [40], the possibility to present a ligand in more than one binding pose and the better treatment of weak or non-binding compounds.

Pharmacophore models for the 4 classes of steroidal compounds possessed some of the features in the published human PXR crystal structures, however the models contained two or three hydrophobic regions (rather than four as shown previously)[27],[28],[31] and one to two hydrogen bond acceptors or a hydrogen bond acceptor and hydrogen bond donor (compared to one hydrogen bond acceptor as shown previously). This might suggest that the steroids evaluated occupy just a part of the ligand binding pocket while larger molecules like rifampicin occupy most of the binding pocket and have subsequently many more interactions with the protein [17]. The addition of the excluded volumes to the pharmacophores was shown to improve the correlation for the training sets and likely acts in a similar manner to using the crystal structures in 5D-QSAR.

Consistent with the QSAR findings were those from docking studies that though modest in success overall, fare much better with individual classes of compounds. The classification was performed using two similarity weighted scoring schemes: one based on a highly potent compound 5α-androstan-3β-ol and the other based on a structurally relevant compound 17β-estradiol. The goal was to test the utility of biasing the scoring scheme with either a structurally relevant compound or a functionally significant compound.

However, in this case 17β-estradiol and 5α-androstan-3β-ol share nearly 75% structural similarity (using MDL Keys and Tanimoto similarity coefficient). The results from the classification studies showed that biasing the scoring scheme with a structurally relevant compound (17β-estradiol) produced classification rates with sensitivity and specificity values averaging at 52% and 50% respectively with slightly better prediction accuracy (Table 1). These results unfortunately cannot be compared with our recent docking study [47] as a different co-crystal ligand was used for the scoring scheme. Although the structure biased scoring scheme performed better among all the compounds, both the scoring schemes performed equally well when individual classes were considered. In the case of androstanes, 6 out of 11 compounds were correctly predicted as activators in docking studies. 5α-Androstan-3β-ol that had the lowest EC_50_ value (described earlier) was predicted to be an activator in all structures. 5α-Androstan-3β-ol binds with very high docking scores and has a hydrogen bond interaction with His407, a key interaction of PXR (Figure 1A). This interaction was consistent among all the androstane activators. However, epitestosterone sulfate has an EC_50_ of 3.39 µM and was misclassified in the combined model using predictions from all structures as a non-activator. Docking studies show that epitestosterone sulfate has a consistently reversed docking pose (when compared with 5α-Androstan-3β-ol) in all the models and the sulfate group is predicted to make a hydrogen bond interaction with His407, as opposed to the steroid ester in 1M13 structure (Figure 1B). A few other misclassified activators were docked in reversed poses and often had favorable hydrogen bonding partners such as sulfates that probably influence the binding mode of these steroids. This is a surprising and novel finding of this study and other researchers should be aware of this when docking similar compounds with this functional group.

Among the bile salts, all four activators were correctly predicted and the ligands bind in a conserved mode with the steroid esters participating in favorable interactions with the side chain of His407 and Arg410, and the steroid rings with hydrophobic groups such as Leu411, Leu239 and Phe281 (Figure 1C). The pregnanes had similar activation patterns as the bile salts and docking studies could predict 4 out of the 9 compounds correctly. Among the misclassified compounds, levonorgestrol was predicted to be an activator in three models, and a non-activator in three models and hence could not be classified with high confidence. Levonorgestrol has an EC_50_ of 4.30 µM and is predicted to have favorable interactions with hPXR as shown in Figure 1D. Despite this, the similarity weighted scoring functions generally performed well in classifying activators as described in the examples above and by the sensitivity values in Table 1. The paucity of available PXR binding data may limit some of the insights from docking experiments performed to date.

It is not surprising that CoMFA and CoMSIA do not perform well as they use rigid alignments of the molecules. This is potentially a seriously limitation given that the binding pocket of PXR may accommodate multiple orientations of the steroids (Figure 1A vs. Figure 1B). Theoretically, 4D- and 5-QSAR should perform better by considering an ensemble of ligand conformations and in fact 4D-QSAR does well within subsets (especially androstanes) but like all methods extrapolates poorly. 5D-QSAR appears to perform the best with the test set. Alignment independent methods like Catalyst which can deal with structurally diverse molecules can generate pharmacophores for the individual classes of compounds but their inter-class predictivity is limited. Another alignment independent method such as using 2D fingerprints and descriptors with the Bayesian classification approach may represent a fast approach to screen for potential PXR agonists, but like all methods their applicability domain [48],[49] is dependent on the training set. In this case the set of steroids would be expected to limit the utility of such models to a relatively narrow class of compounds, although it may be picking up key features in more diverse molecules (Table S3) suggesting overlap in the chemical space.

This study shows the inherent difficulty of producing predictive ligand or structure-based computational models for PXR. Some of the methods used are ligand alignment dependent while others are alignment independent, and each has limitations when used with flexible proteins. These computational models also confirm some of the molecular features (hydrophobicity and hydrogen bond acceptors) identified in previous models and structures, while using a large quantitative dataset to create new QSAR, classification and pharmacophore models to test docking and scoring. The study represents an initial step comparing multiple methods focused on steroidal compounds rather than a more diverse series of drug-like molecules. Using a more diverse series of molecules would have been expected to present even more difficulty for the alignment dependent methods such as CoMFA and CoMSIA. There are also many more commercial computational methods that could be evaluated and compared, although we have used several 3D, 4D, 5D-QSAR methods, machine learning with 2D descriptors, pharmacophore and GOLD docking and scoring methods in this study. The results from these methods could be used in combination as part of a consensus approach or Pareto optimization [50]. The provision of the 115 molecule human PXR dataset is potentially useful as a benchmark PXR set for testing further methods in future. For example, flexible docking methods [51] could be used as well as algorithms that could differentiate multiple binding mechanisms [52].

In conclusion, there are many promiscuous proteins [34] where the modeling of ligand-protein interactions is complicated by a large binding site, multiple binding pockets, protein flexibility or all of the preceding. We have applied several different computational approaches which could also be applied to other proteins like CYPs, transporters and ion channels. This work is therefore more broadly applicable in an attempt to predict whether molecules bind in such flexible proteins, and which methods perform the best. Depending on the desired use of such information, different modeling methods may be appropriate and required. While 2D methods do not encode 3D information like shape [53] they are fast and they can highlight important features likely interacting with the protein. 3D-5D methods provide more shape based information but they are fragile, with a narrow applicability domain and may not be able to differentiate close analogs. Docking is also limited unless key interactions with the protein are already known. Our results suggest that even in the presence of multiple crystal structures, the full range of protein motions may not be captured. As we have previously shown, when docking classification predictions are correct the binding conformation information alone may be instructive [13]. This current analysis indicates that using many different computational approaches (both alignment dependent and alignment independent) may be necessary and expectations should be scaled accordingly if some do not work with such promiscuous proteins. Even with their respective limitations, these methods have provided some useful information of general interest that could be applicable beyond PXR.

## Methods

### Experimental Methodology and Datasets

Human PXR activation was determined by a luciferase-based reporter assay as has been previously described [21],[33],[34]. The datasets modeled in this study were collected by a consistent protocol and have been previously published [31],[54]. Experimental data for four classes of steroidal compounds, namely androstanes, estratrienes, pregnanes and bile acids/ salts are shown in Table S2.

### *In Silico* Methodology: Docking and Scoring

All molecules described in Table S2 were used for docking experiments. The molecules were docked into these six crystallized structures of human PXR (PDB IDs 1M13, 1NRL, 1SKX, 2O9I, 2QNV and one structure co-crystallized with 17β-estradiol that is not in the PDB identified here as EST). In all cases, the crystal structure ligand was removed, and hydrogen atoms were added to the amino acids. All amino acids within 6 Å of the co-crystallized ligand were identified as the binding site. The docking program GOLD (ver 4 [55]) was used for docking all compounds to the binding sites of each PXR crystal structure. GOLD uses genetic algorithm to explore the various conformations of ligands and flexible receptor side chains in the binding pocket. Further, 20 independent docking runs were performed for each ligand. The docked complexes were scored with goldscore [55] and then rescored using similarity weighted scoring scheme (SWscore). For each ligand, the best ranking conformation's goldscore denoted by Si was used to derive the SWscore shown in equation 1. The similarity scores Wi were computed based on 2D similarity encoded in MDL fingerprint keys calculated using Discovery Studio 2.1 (Accelrys, San Diego, CA, USA). The Tanimoto coefficient was used as the metric to compare the molecular fingerprints. The coefficients varied between 0 and 1, where 0 meant maximally dissimilar and 1 coded for maximally similar. The Tanimoto coefficient between fingerprints X and Y has been defined to be: [number of features in intersect (A, B)]/[number of features in union (A,B)], where A and B are two compounds.

So the SWscore is given by, SWscore  =  Wi*Si, where Wi was the similarity score of compound i against 5α-Androstan-3β-ol which had the best EC_50_ value of 0.8 µM for PXR or 17β-estradiol which had a steroid core that was present in most of the compounds. Further, the quality of the scoring function was assessed using standard statistical indicators namely sensitivity (SE), specificity (SP), overall prediction accuracy (Q) and Matthews correlation coefficient (C) (Table 1) and were derived as described previously [22].

### *In Silico* Methodology: Machine Learning with 2D Descriptors

Bayesian models were generated using Discovery Studio 2.1 (Accelrys, San Diego, CA) Laplacian-corrected Bayesian classifier [37],[42],[43],[45],[56]. FCFP_6 fingerprints, AlogP, molecular weight, number of rotatable bonds, number of rings, number of aromatic rings, number of hydrogen bond acceptors, number of hydrogen bond donors and molecular fractional polar surface area were calculated from the input sdf file using the “calculate molecular properties protocol”. The “create Bayesian model protocol” was used for model generation and a custom protocol for validation (leave out 20% 100 times) was used.

### *In Silico* Methodology: 5D-QSAR - Symposar and Raptor

5D-QSAR studies were performed using Raptor [40]. Raptor includes the possibility of representing each ligand molecule as an ensemble of conformations, orientations, stereoisomers and protonation states (4D-QSAR), thereby reducing the bias in identifying the bioactive conformer. In addition, it explicitly allows for induced fit by a dual-shell representation of the three-dimensional binding-site model, onto which the physicochemical properties (hydrophobicity and hydrogen-bonding propensity) are mapped (5D-QSAR). The inner shell is tailored using the most potent ligand of the training set, the outer shell accommodates the topology of all molecules from the training set. The adaptation of both field and topology of the receptor surrogate to each ligand is achieved by combining a steric adjustment to the topology of every ligand and a term due to the attraction or repulsion between ligand and receptor model. The latter is obtained by correlating their physicochemical properties (hydrophobicity and hydrogen-bond propensity) in 3D space. Since the mapping of properties onto the shells is not unambiguously determinable, different models with similar predictive power can be identified. *Raptor* generates a family of receptor models. Such model families may be interpreted to represent the various configuration states of the true biological receptor. The obtained binding affinities are averaged over the individual models.

The underlying scoring function for evaluating ligand-protein interactions includes directional terms for hydrogen bonding (ΔG_Hbond_), hydrophobicity (ΔG_Hphob_) as well as terms for the cost of the topological adaptation (ΔG_IF_) and the changes in entropy (TΔS) upon ligand binding: ΔG_binding_ = ΔG_constant _+ ΔG_Hbond_ + ΔG_HPhob _− TΔS + ΔG_IF_ .

Experimental determination of binding affinity for weak inhibitors is often prevented due to limited solubility or limited sensitivity. Thus, only an upper limit (‘threshold’) for K_i_ values is accessible. To prevent artificial assignment of affinities in a QSAR study including weak binders, the Raptor concept allows the use of a threshold option: the optimization algorithm forces the model to reproduce the binding affinity of the weak- and non-binding ligand molecules to be lower than the experimental limit. Obviously, compounds which are experimentally measured to bind weaker than a threshold K_i_(t) and are correctly classified during the model optimization, no penalty is added to the lack-of-fit value, if, on the other hand, the binding affinity of the ligand is predicted higher than the threshold, the lack-of-fit function applies a penalty proportional to ΔG_binding_(t) − ΔG_binding_.

4D sets of alternative conformations for each ligand as input for Raptor were performed with Symposar [57]. In Symposar the ligand molecules are superimposed onto one or several template molecules, first, on the basis of fuzzy-like 2D substructure similarities and, subsequently, in 3D space with respect to their similarity of physicochemical fields. This two-step process combines the speed of a 2D similarity search with the accuracy and authenticity of protein-ligand interactions in 3D space. The molecules are thereby treated as flexible and are fully relaxed at the end of the alignment process.

## Supporting Information

Table S1 Molecular descriptors for PXR crystal structure ligands calculated with Discovery Studio ver 2.1 (Accelrys, San Diego, CA).
(0.01 MB PDF)

Table S2 Dataset of human PXR activation used for modeling studies.
(0.04 MB PDF)

Table S3 Test set [13] prediction with Bayesian model (activator = EC50<100 µM, non-activator = EC50>100 µM).
(0.02 MB PDF)

Table S4 CoMFA Test Set Predictions
(0.02 MB PDF)

Table S5 To identify which outliers are bringing down the XV-R2 of the CoMFA model, the following table lists the activities as predicted by the cross-validated PLS model: The standard deviation of the residuals in the following table is 0.825, and accordingly, the two possible outliers are highlighted. The two outliers are the only two inactives in the training set.
(0.02 MB PDF)

Table S6 CoMFA outlier analysis.
(0.02 MB PDF)

Table S7 Test set predictions for CoMFA and CoMSIA models.
(0.02 MB PDF)

Table S8 Best model training set correlation (r) values and model statistics (total cost and null cost) for Catalyst Hypogen hypotheses.
(0.01 MB PDF)

Table S9 Three-ordered atom alignments (based on the steroidal core) used in the 4D- QSAR analysis.
(0.01 MB PDF)

Table S10 External Validation test Set Predictions for 4D-QSAR
(0.02 MB PDF)

Table S11 Experimental versus predicted pEC50 values for 115 compounds binding to PXR divided into four different substrate classes - 5D-QSAR.
(0.03 MB PDF)

Text S1 In silico methodology: 3D-QSAR - CoMFA, CoMSIA, In silico methodology: 3D-QSAR - Catalyst, In silico methodology: 4D-QSAR, Supplemental results: CoMFA, CoMSIA and Catalyst. Supplemental data - pharmacophores output files from Discovery Studio Catalyst.
(0.08 MB PDF)

Figure S1 Structural superposition of six PXR crystal structures are shown in ribbon models and colored 1M13 (red), 1NRL (orange), 1SKX (cyan), 2O9I (blue), 2QNV (yellow) and PXR-EST (brown). The co-crystallized ligands are shown as sticks and colored blue for rifampicin, orange for colupulone, dark green for hyperforin, light green for N-{4-[2,2,2-trifluoro-1-hydroxy-1-(trifluromethyl)-ethyl]phenyl}benzenesulfonamide and pink for 17β-estradiol.
(0.68 MB TIF)

Figure S2 CoMFA models for androstanes. A 5α-Androstan-3β-ol (pIC50 = 6.1) shown with the steric component of the CoMFA model. Green denotes areas where steric bulk is favorable for bioactivity while yellow shows areas where steric bulk is not favored. B 5α-Androstan-3β-ol shown with the electrostatic component of the CoMFA model. Blue denotes areas where positive charge is favorable for bioactivity while red shows areas where negative charge is favored.
(0.22 MB TIF)

Figure S3 CoMSIA models for androstanes. A - 17β-dihydroandrosterone (pIC50 = 5.38) with the steric component of the CoMSIA model. Blue denotes areas where steric bulk is favorable for bioactivity while red shows areas where steric bulk is not favored. B 17β-dihydroandrosterone with the hydrophobic component of the CoMSIA model. Purple denotes areas where hydrophobic groups are favorable for bioactivity while grey shows areas where hydrophobic groups are not preferred. C 17β-dihydroandrosterone with the hydrogen bond acceptor component of the CoMSIA model. Blue denotes areas where acceptor groups are favorable for bioactivity while red shows areas where acceptor groups are not preferred.
(0.24 MB TIF)

Figure S4 CoMFA models for pregnanes. A Pregnanedione (pIC50 = 5.59) shown with the steric component of the CoMFA model. Green denotes areas where steric bulk is favorable for bioactivity while yellow shows areas where steric bulk is not favored. B Pregnanedione shown with the electrostatic component of the CoMFA model. Blue denotes areas where positive charge is favorable for bioactivity while red shows areas where negative charge is favored.
(0.26 MB TIF)

Figure S5 CoMSIA models for Pregnanes. A. Inactive training set molecule Pregnenolone Carbonitrile (PCN) (pIC50 = 2.00) with the steric component of the CoMSIA model. Blue denotes areas where steric bulk is favorable for bioactivity while red shows areas where steric bulk is not favored. B Inactive training set molecule PCN shown with the electrostatic component of the CoMSIA model. Blue denotes areas where positive charge is favorable for bioactivity while red shows areas where negative charge is favored. C. Inactive training set molecule PCN with the hydrophobic component of the CoMSIA model. Purple denotes areas where hydrophobic groups are favorable for bioactivity while grey shows areas where hydrophobic groups are not preferred.
(0.23 MB TIF)

Figure S6 A. CoMFA models for bile acids and bile salts. Lithocholic acid acetate (pIC50 = 5.92) shown with the steric component of the CoMFA model. Green denotes areas where steric bulk is favorable for bioactivity while yellow shows areas where steric bulk is not favored. B Lithocholic acid acetate shown with the electrostatic component of the CoMFA model. Blue denotes areas where positive charge is favorable for bioactivity while red shows areas where negative charge is favored.
(0.27 MB TIF)

Figure S7 CoMSIA models of bile acids and bile salts. Using the PLS focused region, CoMSIA components were calculated. A. Hyodeoxycholic acid (pIC50  = 4.42) shown with electrostatic components of the CoMSIA model. Blue denotes areas where positive charge is favorable for bioactivity while red shows areas where negative charge is favored. B. Hyodeoxycholic acid with the hydrophobic component of the CoMSIA model. Purple denotes areas where hydrophobic groups are favorable for bioactivity while grey shows areas where hydrophobic groups are not preferred. C. Hyodeoxycholic acid with the hydrogen bond donor component of the CoMSIA model. Blue denotes areas where donor groups are favorable for bioactivity while red shows areas where donor groups are not preferred.
(0.31 MB TIF)

Figure S8 Catalyst PXR pharmacophores A. Bile acids, B. Estratrienes, C. Androstanes, D Pregnanes pharmacophore features represent Green = hydrogen bond acceptor, purple = hydrogen bond donor, blue = Hydrophobic, gray = excluded volumes.
(0.15 MB TIF)

Figure S9 A. 4D-QSAR for androstanes showing the active conformation of 5α-Androstan-3β-ol, B 4D-QSAR for pregnanes showing the active conformation of pregnanolone, C 4D-QSAR for bile acids/salts showing the active conformation of lithocholic acid acetate.
(0.19 MB TIF)

Figure S10 5D-QSAR Experimental versus predicted pEC50 values for 115 compounds binding to PXR. Training set compounds are displayed in green, test set compounds in red. Threshold compounds are placed at an experimental pEC50 value of 2 to better separate them visually from the other molecules.
(0.11 MB TIF)
