# Supplementary material for: Challenges Predicting Ligand-Receptor Interactions of Promiscuous Proteins: The Nuclear Receptor PXR
Source: PLoS Comput Biol. 2009 Dec 11;5(12):e1000594. doi: 10.1371/journal.pcbi.1000594 (PMC2781111; doi:10.1371/journal.pcbi.1000594)
Supplement: Table S1 — Molecular descriptors for PXR crystal structure ligands calculated with Discovery Studio ver 2.1 (Accelrys, San Diego, CA). (0.01 MB PDF) [file pcbi.1000594.s001.pdf]

Challenges Predicting Ligand-Receptor Interactions of Promiscuous Proteins: The Nuclear Receptor PXR

Sean Ekins[1,2,3*], Sandhya Kortagere[4,] Manisha Iyer[5], Erica J. Reschly[5], Markus A. Lill[6], Matthew R. Redinbo[7,8,9] and Matthew D. Krasowski[5,10].

[1]Collaborations in Chemistry, 601 Runnymede Avenue, Jenkintown, PA 19046, USA

[2]Department of Pharmaceutical Sciences, University of Maryland, 20 Penn Street, Baltimore, MD 21201, USA

[3]Department of Pharmacology, University of Medicine & Dentistry of New Jersey (UMDNJ)-Robert Wood Johnson Medical School, 675 Hoes lane, Piscataway, NJ 08854, USA

[4]Department of Microbiology and Immunology, Drexel University College of Medicine, Philadelphia, PA 19129, USA.

[5]Department of Pathology, University of Pittsburgh, Pittsburgh, PA, 15261,USA

[6]Department of Medicinal Chemistry and Molecular Pharmacology, Purdue University, West Lafaytte, IN 47907, USA.

[7]Department of Chemistry, University of North Carolina at Chapel Hill, Chapel Hill, NC, 27599, USA,

[8]Department of Biochemistry and Biophysics, University of North Carolina at Chapel Hill, Chapel Hill, NC 27599, USA,

[9]The Lineberger Comprehensive Cancer Center, University of North Carolina at Chapel Hill, Chapel Hill, NC 27514, USA,

[10] Current address: Department of Pathology, University of Iowa Hospitals and Clinics, Iowa City, IA 52242, USA

Corresponding author: Sean Ekins, Ph.D., D.Sc., Collaborations in Chemistry, 601 Runnymede Avenue, Jenkintown, PA 19046. Phone 215-687-1320; Fax 215-481-0159;

* Email ekinssean@yahoo.com

Table S1. Molecular descriptors for PXR crystal structure ligands calculated with Discovery Studio ver 2.1 (Accelrys, San Diego, CA). MWT = molecular weight, Rotl Bonds = number of rotatable bonds, HBA = hydrogen bond acceptor, HBD = hydrogen bond donor, Polar surface area = polar surface area.

Compound	ALogP	MWT	Rotl. Bonds	Num_Rings	Num_Aromatic Rings	HBA	HBD	PSA
Estradiol	3.84	272.38	0	4	1	2	2	40.46
T1317	4.84	491.41	8	2	0	3	1	65.99
Colupulone	6.03	400.55	8	1	0	4	2	74.60
Hyperforin	10.11	542.83	13	1	0	4	1	71.44
Rifampicin	3.55	713.81	3	4	1	12	6	201.30
SR12813	4.89	504.53	13	1	1	7	1	110.91
Mean	5.54	487.59	7.50	2.17	0.50	5.33	2.17	94.12
SD	2.41	147.25	5.24	1.47	0.55	3.67	1.94	57.16
