# Supplementary material for: Challenges Predicting Ligand-Receptor Interactions of Promiscuous Proteins: The Nuclear Receptor PXR
Source: PLoS Comput Biol. 2009 Dec 11;5(12):e1000594. doi: 10.1371/journal.pcbi.1000594 (PMC2781111; doi:10.1371/journal.pcbi.1000594)
Supplement: Table S2 — Dataset of human PXR activation used for modeling studies. (0.04 MB PDF) [file pcbi.1000594.s002.pdf]

Challenges Predicting Ligand-Receptor Interactions of Promiscuous Proteins: The Nuclear Receptor PXR

Sean Ekins[1,2,3*], Sandhya Kortagere[4,] Manisha Iyer[5], Erica J. Reschly[5], Markus A. Lill[6], Matthew R. Redinbo[7,8,9] and Matthew D. Krasowski[5,10].

[1]Collaborations in Chemistry, 601 Runnymede Avenue, Jenkintown, PA 19046, USA

[2]Department of Pharmaceutical Sciences, University of Maryland, 20 Penn Street, Baltimore, MD 21201, USA

[3]Department of Pharmacology, University of Medicine & Dentistry of New Jersey (UMDNJ)-Robert Wood Johnson Medical School, 675 Hoes lane, Piscataway, NJ 08854, USA

[4]Department of Microbiology and Immunology, Drexel University College of Medicine, Philadelphia, PA 19129, USA.

[5]Department of Pathology, University of Pittsburgh, Pittsburgh, PA, 15261,USA

[6]Department of Medicinal Chemistry and Molecular Pharmacology, Purdue University, West Lafaytte, IN 47907, USA.

[7]Department of Chemistry, University of North Carolina at Chapel Hill, Chapel Hill, NC, 27599, USA,

[8]Department of Biochemistry and Biophysics, University of North Carolina at Chapel Hill, Chapel Hill, NC 27599, USA,

[9]The Lineberger Comprehensive Cancer Center, University of North Carolina at Chapel Hill, Chapel Hill, NC 27514, USA,

[10] Current address: Department of Pathology, University of Iowa Hospitals and Clinics, Iowa City, IA 52242, USA

Corresponding author: Sean Ekins, Ph.D., D.Sc., Collaborations in Chemistry, 601 Runnymede Avenue, Jenkintown, PA 19046. Phone 215-687-1320; Fax 215-481-0159;

* Email ekinssean@yahoo.com

Table S2. Dataset of human PXR activation used for modeling studies and classification based on docking experiments.

Compound Name	Human PXR EC_{50} (μM)	Human PXR Activity ($-\log EC_{50}$)	Human PXR Efficacy (ε)	Test set for ComFA	Docking Classification
ANDROSTANES					
17β-dihydroandrosterone	4.15	5.38	0.68		A
Androstanedione	12.7	4.90	0.87		N
Androstanol	6.27	5.20	0.5		A
Androsterone	18.6	4.73	0.93		A
Dihydrotestosterone	11.4	4.94	0.39		A
Etiocholanolone	5.7	5.24	0.54		A
Androstenedione	20.6	4.69	0.59	X	N
Testosterone	71.9	4.14	0.22		A
DHEA	32.5	4.49	0.52		A
Androstenol	5.5	5.26	0.7		A
11β-Hydroxyetiocholanolone	19.2	4.72	0.51		A
DHEA sulfate (sodium salt)	48.2	4.32	0.22		N
Epiandrosterone	4.91	5.31	0.7		A
11-Ketoetiocholanone	41.1	4.39	0.15	X	A
Epitestosterone	68	4.17	0.9		A
Epitestosterone glucuronide	13.8	4.86	0.69		N
Epitestosterone sulfate	3.39	5.47	0.67	X	N
Etiocholanolone glucuronide	10000	2.00	0		N
5α-Androstane	10000	2.00	0	X	N
5α-Androstan-3β-ol	0.8	6.10	0.43		A
16,(5α)-Androsten-3β-ol	4.77	5.32	1.01		A
16,(5α)-Androsten-3-one	3	5.52	0.96		A
5β-Androstan-3α-ol	1.41	5.85	1.12		A
4,16-Androstadien-3-one	7.1	5.15	0.64	X	N
5,16-Androstadien-3β-ol	10000	2.00	0		A
PREGNANES					
Pregnanediol	5.1	5.29	0.34		A
Pregnanedione	2.6	5.59	0.97		A
Corticosterone	9.95	5.00	0.54	X	N
Cortexolone	23	4.64	0.49		N
Aldosterone	55	4.26	0.21		N
Cortisone	69.4	4.16	0.28	X	N
Progesterone	14.7	4.83	0.57		N

17-Hydroxyprogesterone	17.7	4.75	0.7		A
Cortexone	2.44	5.61	0.3		N
Cortisol	47.6	4.32	0.66		N
17-Hydroxypregnenolone	33.5	4.47	0.36		A
Pregnenolone	2.3	5.64	1.26	X	A
Pregnenolone carbonitrile (PCN)	10000	2.00	0		N
Allopregnanolone	4.21	5.38	0.46		A
Allopregnanediol	52.5	4.28	0.16		A
Pregnanediol glucuronide	55.1	4.26	0.17	X	N
Cortol	47.2	4.33	0.83		N
Cortolone	44.7	4.35	0.72		N
Tetrahydrocortisone	52.7	4.28	0.8		N
THDOC	12.5	4.90	0.26		N
17α,20β-Dihydroxyprogesterone	10000	2.00	0	X	N
17α,20β-Dihydroxyprogesterone sodium sulfate	1.88	5.73	0.66		N
5β-Pregnan-3α,20β,diol	3.81	5.42	0.49		A
Tetrahydrocortisol	46.5	4.33	0.73		N
Pregnanolone	10.4	4.98	0.55		A
Pregnenolone sulfate	10000	2.00	0		N
Levonorgestrol	4.30	5.37	0.35		N
Norethindrone	25.60	4.59	0.32		A
Dexamethasone	41.00	4.39	0.83	X	N
ESTRATRIENES					
Estradiol	16	4.80	0.34		A
Estrone	37.9	4.42	0.47		N
Estriol	10000	2.00	0		N
16α-Hydroxyestrone	2.5	5.60	0.42		N
4-Methoxyestrone	4	5.40	0.93		N
Estetrol	2.14	5.67	0.29		N
2-Hydroxyestrone	3.6	5.44	0.93		N
Estrone 3-sulfate	3.4	5.47	0.43		N
Estradiol glucuronide	10000	2.00	0		N
Estradiol 3-sulfate	0.89	6.05	0.6		N
Ethinyl estradiol	1.9	5.72	0.68		A
BILE ACIDS /SALTS					
5β-Petromyzonol	10000	2.00	0		A
Hyodeoxycholic acid	38.1	4.42	0.17		A
Taurohyodeoxycholic acid	19	4.72	0.39		N
Murocholic acid (Murideoxycholic acid)	10000	2.00	0		A
Chenodeoxycholic acid	10000	2.00	0	X	A
Glycochenodeoxycholic acid	10000	2.00	0		N

Taurochenodeoxycholic acid	104	3.98	0.5		N
Deoxycholic acid	50.2	4.30	0.19		A
Glycodeoxycholic acid	10000	2.00	0	X	N
Taurodeoxycholic acid	10000	2.00	0		N
Lithocholic acid	10	5.00	0.15		A
Glycolithocholic acid	16.1	4.79	0.49		A
Taurolithocholic acid	19.8	4.70	0.15		N
7,12-Diketolithocholic acid	35.5	4.45	0.31		A
7-Ketolithocholic acid	21.5	4.67	0.58		A
12-Ketolithocholic acid	31.3	4.50	0.86		A
ω-Muricholic acid	10000	2.00	0		A
α-Muricholic acid	56	4.25	0.79		A
β-Muricholic acid	10000	2.00	0		A
Cholic acid	11.6	4.94	0.56		A
Glycocholic acid	10000	2.00	0		N
Taurocholic acid	10000	2.00	0	X	N
5β-cholestan-3α,7α,12α-triol	10000	2.00	0	X	A
20α-hydroxycholesterol	10000	2.00	0		A
Cholesterol	10000	2.00	0		A
23-Nordeoxycholic acid	16.4	4.79	0.5	X	A
23-Norcholic acid	96	4.02	0.49		A
3-Ketopetromyzonol	10000	2.00	0		A
Petromyzonol	10000	2.00	0		A
Petromyzonol sulfate	28.1	4.55	1.02	X	N
Allocholic acid	10000	2.00	0		A
3-Ketopetromyzonol sulfate	10000	2.00	0		N
3-Keto-7α,12α-dihydroxy-5α-cholanic acid	12.1	4.92	0.26		A
Scymnol - sulfated	49.3	4.31	0.39	X	N
Scymnol - desulfated	10000	2.00	0		A
5α-cyprinol - sulfated	25.9	4.59	1.1		N
5α-cyprinol - desulfated	10000	2.00	0		A
Alligator bile acid	10000	2.00	0		A
Myxinol - sulfated	10000	2.00	0		A
Myxinol - desulfated	10000	2.00	0		A
Lithocholic acid 3-sulfate	117.9	3.93	0.69		N
7-Ketodeoxycholic acid	58.2	4.24	0.49		A
Glycolithocholic acid 3-sulfate, disodium salt	56.2	4.25	0.19		N
Taurolithocholic acid 3-sulfate, disodium salt	83.2	4.08	0.55	X	N
α-Cholestanol	10000	2.00	0	X	A
Tauro-β-muricholic acid	10000	2.00	0		N
7α-Hydroxycholesterol	10000	2.00	0		A
3-Ketolithocholic acid	8.3	5.08	0.42		A

Lithocholic acid acetate	1.2	5.92	0.54		A
Lithocholic acid acetate methyl ester	1.1	5.96	0.49		A
