# Supplementary material for: Challenges Predicting Ligand-Receptor Interactions of Promiscuous Proteins: The Nuclear Receptor PXR
Source: PLoS Comput Biol. 2009 Dec 11;5(12):e1000594. doi: 10.1371/journal.pcbi.1000594 (PMC2781111; doi:10.1371/journal.pcbi.1000594)
Supplement: Table S3 — Test set [13] prediction with Bayesian model (activator = EC50<100 µM, non-activator = EC50>100 µM). (0.02 MB PDF) [file pcbi.1000594.s003.pdf]

Challenges Predicting Ligand-Receptor Interactions of Promiscuous Proteins: The Nuclear Receptor PXR

Sean Ekins[1,2,3*], Sandhya Kortagere[4,] Manisha Iyer[5], Erica J. Reschly[5], Markus A. Lill[6], Matthew R. Redinbo[7,8,9] and Matthew D. Krasowski[5,10].

[1]Collaborations in Chemistry, 601 Runnymede Avenue, Jenkintown, PA 19046, USA

[2]Department of Pharmaceutical Sciences, University of Maryland, 20 Penn Street, Baltimore, MD 21201, USA

[3]Department of Pharmacology, University of Medicine & Dentistry of New Jersey (UMDNJ)-Robert Wood Johnson Medical School, 675 Hoes lane, Piscataway, NJ 08854, USA

[4]Department of Microbiology and Immunology, Drexel University College of Medicine, Philadelphia, PA 19129, USA.

[5]Department of Pathology, University of Pittsburgh, Pittsburgh, PA, 15261,USA

[6]Department of Medicinal Chemistry and Molecular Pharmacology, Purdue University, West Lafaytte, IN 47907, USA.

[7]Department of Chemistry, University of North Carolina at Chapel Hill, Chapel Hill, NC, 27599, USA,

[8]Department of Biochemistry and Biophysics, University of North Carolina at Chapel Hill, Chapel Hill, NC 27599, USA,

[9]The Lineberger Comprehensive Cancer Center, University of North Carolina at Chapel Hill, Chapel Hill, NC 27514, USA,

[10] Current address: Department of Pathology, University of Iowa Hospitals and Clinics, Iowa City, IA 52242, USA

Corresponding author: Sean Ekins, Ph.D., D.Sc., Collaborations in Chemistry, 601 Runnymede Avenue, Jenkintown, PA 19046. Phone 215-687-1320; Fax 215-481-0159;

* Email ekinssean@yahoo.com

Table S3. Test set [7] prediction with Bayesian model (activator = EC_{50} < 100 μM, non-activiator = EC_{50} > 100 μM).

Molecule	classification	PXR n119 FCFP6 BayesianTempModel
Morphine	n	9.873
Oxycodone	n	8.986
Cdd3508	a	8.873
Nabumetone	a	8.454
Reserpine	a	8.018
Venlafaxine	n	7.649
Doxepin	n	7.553
Amitriptyline	n	7.544
Naloxone	a	7.43
Zolpidem	a	7.271
Diltiazem	a	6.722
Oxcarbazepine	a	5.724
Midazolam	a	5.706
Triazolam	a	5.677
Efavirenz	a	5.668
Omeprazole	a	5.455
Carbamazepine	a	5.369
Naproxen	n	5.29
Cdd3501	a	5.266
Ondansetron	a	5.166
Terbinafine	a	4.985
Cdd3536	a	4.978
Tacrine	n	4.935
Cdd3543	a	4.766
Cdd3538	a	4.766
Phenylbutazone	a	4.735
Raloxifene	a	4.54
Metoprolol	n	4.434
Ibuprofen	n	4.395
C2ba-11	n	4.372
Cdd3540	a	4.367
Bergamottin	a	4.357
Loratadine	a	4.284
Acetaminophen	n	4.176
Ketoconazole	n	4.151
C2ba-12	n	4.096
Cdd3530	a	4.053
Cdd3532	a	4.053
C2ba-6	a	3.914
Rosiglitazone	a	3.886

C2ba-10	a	3.757
Mycophenolic Acid	n	3.686
Theophylline	n	3.626
Gemfibrozil	n	3.496
Nevirapine	n	3.402
Pioglitazone	a	3.394
Flurbiprofen	a	3.303
C2ba-7	a	3.283
Paroxetine	n	3.24
Haloperidol	a	3.097
Diclofenac	a	3.087
Chlorpromazine	n	3.069
Desipramine	n	2.702
Montelukast Sodium	a	2.7
Lansoprazole	a	2.448
Fluconazole	n	2.401
Rifapentine	a	2.36
Simvastatin	a	2.255
Dapsone	n	2.143
C2ba-248	a	2.085
Meclizine	a	1.961
C2ba-8	a	1.922
Tolmetin	n	1.642
Sildenafil	a	1.623
Meloxicam	a	1.528
C2ba-9	n	1.362
Levofloxacin	n	1.353
Lamotrigine	n	1.255
Rabeprazole Sodium	a	1.223
Risperidone	n	1.192
Tocainide	n	1.172
Flutamide	a	1.035
Doxorubicin	n	0.922
Rifabutin	a	0.919
Felodipine	a	0.858
Albuterol	n	0.805
Etodolac	n	0.777
Celecoxib	a	0.75
Bupropion	a	0.684
Nicardipine	a	0.629
Zomitriptan	n	0.493
Atenolol	n	0.49
Metronidazole	n	0.415
Topotecan	a	0.219
Valsartan	n	0.056
C2ba-3	n	0.043

C2ba-13	a	-0.13
Nadolol	n	-0.137
Quinapril	a	-0.206
Terazosin	n	-0.21
C2ba-251	a	-0.327
Propranolol	n	-0.489
Amlodipine	a	-1.032
Enalapril	n	-1.062
Doxazosin	n	-1.089
Sumatriptan	n	-1.557
Etoposide	a	-1.731
Isotretinoin	a	-1.816
Ranitidine	n	-1.875
Glimepiride	a	-2.077
Mevastatin	a	-2.279
Timolol	n	-2.286
Azithromycin	n	-2.459
Ethosuximide	n	-2.648
Forskolin	a	-2.905
Valproic Acid	a	-3.144
C2ba-5	a	-3.159
Linezolid	n	-3.175
Captopril		-3.35
Famotidine	n	-3.39
Ciprofloxacin	n	-3.513
Furosemide	n	-3.686
Fexofenadine	n	-3.892
Erythromycin	n	-3.939
Clarithromycin	n	-3.962
1-9-Dideoxydorskolin	a	-3.975
Fluvastatin	n	-4.252
Cyclophosphamide	a	-4.665
Cimetidine	n	-5.13
Budesonide	a	-5.486
Acyclovir	n	-5.508
Lisinopril	n	-5.702
Cholecalciferol	n	-14.308
