# Supplementary material for: Challenges Predicting Ligand-Receptor Interactions of Promiscuous Proteins: The Nuclear Receptor PXR
Source: PLoS Comput Biol. 2009 Dec 11;5(12):e1000594. doi: 10.1371/journal.pcbi.1000594 (PMC2781111; doi:10.1371/journal.pcbi.1000594)
Supplement: Table S4 — CoMFA Test Set Predictions (0.02 MB PDF) [file pcbi.1000594.s004.pdf]

Challenges Predicting Ligand-Receptor Interactions of Promiscuous Proteins: The Nuclear Receptor PXR

Sean Ekins[1,2,3*], Sandhya Kortagere[4,] Manisha Iyer[5], Erica J. Reschly[5], Markus A. Lill[6], Matthew R. Redinbo[7,8,9] and Matthew D. Krasowski[5,10].

[1]Collaborations in Chemistry, 601 Runnymede Avenue, Jenkintown, PA 19046, USA

[2]Department of Pharmaceutical Sciences, University of Maryland, 20 Penn Street, Baltimore, MD 21201, USA

[3]Department of Pharmacology, University of Medicine & Dentistry of New Jersey (UMDNJ)-Robert Wood Johnson Medical School, 675 Hoes lane, Piscataway, NJ 08854, USA

[4]Department of Microbiology and Immunology, Drexel University College of Medicine, Philadelphia, PA 19129, USA.

[5]Department of Pathology, University of Pittsburgh, Pittsburgh, PA, 15261,USA

[6]Department of Medicinal Chemistry and Molecular Pharmacology, Purdue University, West Lafaytte, IN 47907, USA.

[7]Department of Chemistry, University of North Carolina at Chapel Hill, Chapel Hill, NC, 27599, USA,

[8]Department of Biochemistry and Biophysics, University of North Carolina at Chapel Hill, Chapel Hill, NC 27599, USA,

[9]The Lineberger Comprehensive Cancer Center, University of North Carolina at Chapel Hill, Chapel Hill, NC 27514, USA,

[10] Current address: Department of Pathology, University of Iowa Hospitals and Clinics, Iowa City, IA 52242, USA

Corresponding author: Sean Ekins, Ph.D., D.Sc., Collaborations in Chemistry, 601 Runnymede Avenue, Jenkintown, PA 19046. Phone 215-687-1320; Fax 215-481-0159;

* Email ekinssean@yahoo.com

Table S4 – CoMFA Test Set Predictions

	Observed. Activity	Predicted Activity
Androstenedione	4.69	5.06
11-Ketoetiocholanone	4.39	5.19
Epitestosterone sulfate	5.47	3.97
5α-Androstane	2.00	5.71
4,16-Androstadien-3-one	5.15	5.38
Corticosterone	5.00	4.82
Cortisone	4.16	2.92
Pregnenolone	5.64	3.42
Pregnanediol glucuronide	4.26	4.64
17α,20β-Dihydroxyprogesterone	2.00	5.35
Dexamethasone	4.39	5.23
Chenodeoxycholic acid	2.00	4.08
Glycodeoxycholic acid	2.00	3.13
Taurocholic acid	2.00	2.06
5β-cholestan-3α,7α,12α,-triol	2.00	2.94
23-Nordeoxycholic acid	4.79	4.55
Petromyzonol sulfate	4.55	1.41
Scymnol – sulfated	4.31	2.70
Taurolithocholic acid 3-sulfate, disodium salt	4.08	5.14
α-Cholestanol	2.00	3.50
