# Supplementary material for: Challenges Predicting Ligand-Receptor Interactions of Promiscuous Proteins: The Nuclear Receptor PXR
Source: PLoS Comput Biol. 2009 Dec 11;5(12):e1000594. doi: 10.1371/journal.pcbi.1000594 (PMC2781111; doi:10.1371/journal.pcbi.1000594)
Supplement: Table S5 — To identify which outliers are bringing down the XV-R2 of the CoMFA model, the following table lists the activities as predicted by the cross-validated PLS model: The standard deviation of the residuals in the following table is 0.825, and accordingly, the two possible outliers are highlighted. The two outliers are the only two inactives in the training set. (0.02 MB PDF) [file pcbi.1000594.s005.pdf]

Challenges Predicting Ligand-Receptor Interactions of Promiscuous Proteins: The Nuclear Receptor PXR

Sean Ekins[1,2,3*], Sandhya Kortagere[4,] Manisha Iyer[5], Erica J. Reschly[5], Markus A. Lill[6], Matthew R. Redinbo[7,8,9] and Matthew D. Krasowski[5,10].

[1]Collaborations in Chemistry, 601 Runnymede Avenue, Jenkintown, PA 19046, USA

[2]Department of Pharmaceutical Sciences, University of Maryland, 20 Penn Street, Baltimore, MD 21201, USA

[3]Department of Pharmacology, University of Medicine & Dentistry of New Jersey (UMDNJ)-Robert Wood Johnson Medical School, 675 Hoes lane, Piscataway, NJ 08854, USA

[4]Department of Microbiology and Immunology, Drexel University College of Medicine, Philadelphia, PA 19129, USA.

[5]Department of Pathology, University of Pittsburgh, Pittsburgh, PA, 15261,USA

[6]Department of Medicinal Chemistry and Molecular Pharmacology, Purdue University, West Lafaytte, IN 47907, USA.

[7]Department of Chemistry, University of North Carolina at Chapel Hill, Chapel Hill, NC, 27599, USA,

[8]Department of Biochemistry and Biophysics, University of North Carolina at Chapel Hill, Chapel Hill, NC 27599, USA,

[9]The Lineberger Comprehensive Cancer Center, University of North Carolina at Chapel Hill, Chapel Hill, NC 27514, USA,

[10] Current address: Department of Pathology, University of Iowa Hospitals and Clinics, Iowa City, IA 52242, USA

Corresponding author: Sean Ekins, Ph.D., D.Sc., Collaborations in Chemistry, 601 Runnymede Avenue, Jenkintown, PA 19046. Phone 215-687-1320; Fax 215-481-0159;

* Email ekinssean@yahoo.com

Table S5. To identify which outliers are bringing down the XV-R^2 of the CoMFA model, the following table lists the activities as predicted by the cross-validated PLS model: The standard deviation of the residuals in the following table is 0.825, and accordingly, the two possible outliers are highlighted in bold. The two outliers are the only two inactives in the training set.

		Exp. Activity	Pred. Activity	Residual
AN01	17β-dihydroandrosterone	5.38	5.49	0.108
AN02	Androstanedione	4.90	5.13	0.234
AN03	Androstanol	5.20	5.43	0.227
AN04	Androsterone	4.73	4.71	-0.020
AN05	Dihydrotestosterone	4.94	5.24	0.297
AN06	Etiocholanolone	5.24	4.79	-0.454
AN08	Testosterone	4.14	4.42	0.277
AN09	DHEA	4.49	3.51	-0.978
AN10	Androstenol	5.26	4.87	-0.390
AN11	11β-Hydroxyetiocholanolone	4.72	5.42	0.703
AN12	DHEA sulfate (sodium salt)	4.32	4.65	0.333
AN13	Epiandrosterone	5.31	5.11	-0.199
AN15	Epitestosterone	4.17	3.75	-0.417
AN16	Epitestosterone glucuronide	4.86	4.71	-0.150
AN18	*Etiocholanolone glucuronide*	**2.00**	***4.19***	***2.190***
AN20	5α-Androstan-3β-ol	6.10	5.56	-0.537
AN21	16,(5α)-Androsten-3β-ol	5.32	6.08	0.759
AN22	16,(5α)-Androsten-3-one	5.52	5.15	-0.373
AN23	5β-Androstan-3α-ol	5.85	5.27	-0.581
AN25	*5,16-Androstadien-3β-ol*	**2.00**	***4.21***	***2.210***
