# Supplementary material for: Challenges Predicting Ligand-Receptor Interactions of Promiscuous Proteins: The Nuclear Receptor PXR
Source: PLoS Comput Biol. 2009 Dec 11;5(12):e1000594. doi: 10.1371/journal.pcbi.1000594 (PMC2781111; doi:10.1371/journal.pcbi.1000594)
Supplement: Table S6 — CoMFA outlier analysis. (0.02 MB PDF) [file pcbi.1000594.s006.pdf]

Challenges Predicting Ligand-Receptor Interactions of Promiscuous Proteins: The Nuclear Receptor PXR

Sean Ekins[1,2,3*], Sandhya Kortagere[4,] Manisha Iyer[5], Erica J. Reschly[5], Markus A. Lill[6], Matthew R. Redinbo[7,8,9] and Matthew D. Krasowski[5,10].

[1]Collaborations in Chemistry, 601 Runnymede Avenue, Jenkintown, PA 19046, USA

[2]Department of Pharmaceutical Sciences, University of Maryland, 20 Penn Street, Baltimore, MD 21201, USA

[3]Department of Pharmacology, University of Medicine & Dentistry of New Jersey (UMDNJ)-Robert Wood Johnson Medical School, 675 Hoes lane, Piscataway, NJ 08854, USA

[4]Department of Microbiology and Immunology, Drexel University College of Medicine, Philadelphia, PA 19129, USA.

[5]Department of Pathology, University of Pittsburgh, Pittsburgh, PA, 15261,USA

[6]Department of Medicinal Chemistry and Molecular Pharmacology, Purdue University, West Lafaytte, IN 47907, USA.

[7]Department of Chemistry, University of North Carolina at Chapel Hill, Chapel Hill, NC, 27599, USA,

[8]Department of Biochemistry and Biophysics, University of North Carolina at Chapel Hill, Chapel Hill, NC 27599, USA,

[9]The Lineberger Comprehensive Cancer Center, University of North Carolina at Chapel Hill, Chapel Hill, NC 27514, USA,

[10] Current address: Department of Pathology, University of Iowa Hospitals and Clinics, Iowa City, IA 52242, USA

Corresponding author: Sean Ekins, Ph.D., D.Sc., Collaborations in Chemistry, 601 Runnymede Avenue, Jenkintown, PA 19046. Phone 215-687-1320; Fax 215-481-0159;

* Email ekinssean@yahoo.com

Table S6 CoMFA outlier analysis (Bold = outliers).

		Exp. Activity	Pred. Activity	Residual
PR1	Pregnanediol	5.29	5.67	0.378
PR2	Pregnanedione	5.59	5.36	-0.225
PR4	Cortexolone	4.64	5.09	0.452
PR5	Aldosterone	4.26	4.74	0.480
PR7	Progesterone	4.83	5.08	0.247
PR8	17-Hydroxyprogesterone	4.75	4.91	0.158
PR9	Cortexone	5.61	4.64	-0.973
PR10	Cortisol	4.32	4.49	0.168
PR11	*17-Hydroxypregnenolone*	**4.47**	***2.47***	***-2.005***
PR13	Pregnenolone carbonitrile (PCN)	**2.00**	***3.47***	***1.470***
PR14	Allopregnanolone	5.38	4.52	-0.856
PR15	Allopregnanediol	4.28	5.01	0.730
PR17	Cortol	4.33	4.71	0.384
PR18	Cortolone	4.35	4.18	-0.170
PR19	Tetrahydrocortisone	4.28	4.26	-0.018
PR20	THDOC	4.90	4.78	-0.123
PR22	17α,20β-Dihydroxyprogesterone sodium sulfate	5.73	5.07	-0.656
PR23	5β-Pregnan-3α,20β,diol	5.42	5.48	0.061
PR24	Tetrahydrocortisol	4.33	4.10	-0.233
PR25	Pregnanolone	4.98	5.04	0.057
PR26	Pregnenolone sulfate	2.00	2.79	0.790
PR27	Levonorgestrol	5.37	6.11	0.743
PR28	Norethindrone	4.59	4.14	-0.452
