# Supplementary material for: Challenges Predicting Ligand-Receptor Interactions of Promiscuous Proteins: The Nuclear Receptor PXR
Source: PLoS Comput Biol. 2009 Dec 11;5(12):e1000594. doi: 10.1371/journal.pcbi.1000594 (PMC2781111; doi:10.1371/journal.pcbi.1000594)
Supplement: Table S7 — Test set predictions for CoMFA and CoMSIA models. (0.02 MB PDF) [file pcbi.1000594.s007.pdf]

Challenges Predicting Ligand-Receptor Interactions of Promiscuous Proteins: The Nuclear Receptor PXR

Sean Ekins[1,2,3*], Sandhya Kortagere[4,] Manisha Iyer[5], Erica J. Reschly[5], Markus A. Lill[6], Matthew R. Redinbo[7,8,9] and Matthew D. Krasowski[5,10].

[1]Collaborations in Chemistry, 601 Runnymede Avenue, Jenkintown, PA 19046, USA

[2]Department of Pharmaceutical Sciences, University of Maryland, 20 Penn Street, Baltimore, MD 21201, USA

[3]Department of Pharmacology, University of Medicine & Dentistry of New Jersey (UMDNJ)-Robert Wood Johnson Medical School, 675 Hoes lane, Piscataway, NJ 08854, USA

[4]Department of Microbiology and Immunology, Drexel University College of Medicine, Philadelphia, PA 19129, USA.

[5]Department of Pathology, University of Pittsburgh, Pittsburgh, PA, 15261,USA

[6]Department of Medicinal Chemistry and Molecular Pharmacology, Purdue University, West Lafaytte, IN 47907, USA.

[7]Department of Chemistry, University of North Carolina at Chapel Hill, Chapel Hill, NC, 27599, USA,

[8]Department of Biochemistry and Biophysics, University of North Carolina at Chapel Hill, Chapel Hill, NC 27599, USA,

[9]The Lineberger Comprehensive Cancer Center, University of North Carolina at Chapel Hill, Chapel Hill, NC 27514, USA,

[10] Current address: Department of Pathology, University of Iowa Hospitals and Clinics, Iowa City, IA 52242, USA

Corresponding author: Sean Ekins, Ph.D., D.Sc., Collaborations in Chemistry, 601 Runnymede Avenue, Jenkintown, PA 19046. Phone 215-687-1320; Fax 215-481-0159;

* Email ekinssean@yahoo.com

Table S7. Test set predictions for CoMFA (R^2 = 0.11) and CoMSIA (R^2 = 0.04) models (Bold = outliers).

Compound	Experimental Activity	Predicted Activity (CoMFA)	Predicted Activity (CoMSIA)
ANDROSTANES			
Androstenedione	4.69	4.84	4.74
11-Ketoetiocholanone	4.39	5.38	5.17
Epitestosterone sulfate	5.47	4.17	3.95
5α-Androstane	**2.00**	**5.49**	**5.85**
4,16-Androstadien-3-one	5.15	5.87	5.84
PREGNANES			
Corticosterone	5.00	4.56	4.48
Cortisone	4.16	5.17	4.81
Pregnenolone	5.64	5.13	5.08
Pregnanediol glucuronide	4.26	2.42	2.24
17α,20β-Dihydroxyprogesterone	**2.00**	**5.57**	**5.15**
Dexamethasone	4.39	3.69	3.82
BILE ACIDS / SALTS			
Chenodeoxycholic acid	**2.00**	**4.02**	**4.27**
Glycodeoxycholic acid	**2.00**	2.95	3.67
Taurocholic acid	2.00	2.35	1.92
5β-cholestan-3α,7α,12α,-triol	2.00	2.00	2.58
23-Nordeoxycholic acid	4.79	4.66	4.03
Petromyzonol sulfate	4.55	2.26	2.14
Scymnol – sulfated	**4.31**	**2.55**	**2.21**
Taurolithocholic acid 3-sulfate, disodium salt	4.08	4.26	4.02
α-Cholestanol	2.00	2.04	2.58
