# Supplementary material for: Challenges Predicting Ligand-Receptor Interactions of Promiscuous Proteins: The Nuclear Receptor PXR
Source: PLoS Comput Biol. 2009 Dec 11;5(12):e1000594. doi: 10.1371/journal.pcbi.1000594 (PMC2781111; doi:10.1371/journal.pcbi.1000594)
Supplement: Table S8 — Best model training set correlation (r) values and model statistics (total cost and null cost) for Catalyst Hypogen hypotheses. (0.01 MB PDF) [file pcbi.1000594.s008.pdf]

Challenges Predicting Ligand-Receptor Interactions of Promiscuous Proteins: The Nuclear Receptor PXR

Sean Ekins[1,2,3*], Sandhya Kortagere[4,] Manisha Iyer[5], Erica J. Reschly[5], Markus A. Lill[6], Matthew R. Redinbo[7,8,9] and Matthew D. Krasowski[5,10].

[1]Collaborations in Chemistry, 601 Runnymede Avenue, Jenkintown, PA 19046, USA

[2]Department of Pharmaceutical Sciences, University of Maryland, 20 Penn Street, Baltimore, MD 21201, USA

[3]Department of Pharmacology, University of Medicine & Dentistry of New Jersey (UMDNJ)-Robert Wood Johnson Medical School, 675 Hoes lane, Piscataway, NJ 08854, USA

[4]Department of Microbiology and Immunology, Drexel University College of Medicine, Philadelphia, PA 19129, USA.

[5]Department of Pathology, University of Pittsburgh, Pittsburgh, PA, 15261,USA

[6]Department of Medicinal Chemistry and Molecular Pharmacology, Purdue University, West Lafaytte, IN 47907, USA.

[7]Department of Chemistry, University of North Carolina at Chapel Hill, Chapel Hill, NC, 27599, USA,

[8]Department of Biochemistry and Biophysics, University of North Carolina at Chapel Hill, Chapel Hill, NC 27599, USA,

[9]The Lineberger Comprehensive Cancer Center, University of North Carolina at Chapel Hill, Chapel Hill, NC 27514, USA,

[10] Current address: Department of Pathology, University of Iowa Hospitals and Clinics, Iowa City, IA 52242, USA

Corresponding author: Sean Ekins, Ph.D., D.Sc., Collaborations in Chemistry, 601 Runnymede Avenue, Jenkintown, PA 19046. Phone 215-687-1320; Fax 215-481-0159;

* Email ekinssean@yahoo.com

Table S8. Best model training set correlation (r) values and model statistics (total cost and null cost) for Catalyst Hypogen hypotheses.

Compounds	Without excluded volumes	2 excluded volumes	Variable weight/tolerance	2 excluded volumes and variable weight/ tolerance
Bile Acids	0.65 (302.4 – 371.9)	0.79 (272.5-371.9)	0.71 (314.0-371.9)	0.81 (294.5-371.9)
Estratrienes	0.84 (73.9-82.8)	0.86 (66.9-82.8)	0.84 (96.3-82.8)	0.92 (89.5-82.8)
Androstanes	0.75 (127.7-149.2)	0.75 (127.7-149.2)	0.83 (141.7-149.2)	0.83 (141.7-149.2)
Pregnanes	0.62 (150.2-159.6)	0.81 (141.9-159.6)	0.83 (159.2-159.6)	0.92 (153 – 159.6)
