# Supplementary material for: Challenges Predicting Ligand-Receptor Interactions of Promiscuous Proteins: The Nuclear Receptor PXR
Source: PLoS Comput Biol. 2009 Dec 11;5(12):e1000594. doi: 10.1371/journal.pcbi.1000594 (PMC2781111; doi:10.1371/journal.pcbi.1000594)
Supplement: Table S10 — External Validation test Set Predictions for 4D-QSAR (0.02 MB PDF) [file pcbi.1000594.s010.pdf]

Challenges Predicting Ligand-Receptor Interactions of Promiscuous Proteins: The Nuclear Receptor PXR

Sean Ekins[1,2,3*], Sandhya Kortagere[4,] Manisha Iyer[5], Erica J. Reschly[5], Markus A. Lill[6], Matthew R. Redinbo[7,8,9] and Matthew D. Krasowski[5,10].

[1]Collaborations in Chemistry, 601 Runnymede Avenue, Jenkintown, PA 19046, USA

[2]Department of Pharmaceutical Sciences, University of Maryland, 20 Penn Street, Baltimore, MD 21201, USA

[3]Department of Pharmacology, University of Medicine & Dentistry of New Jersey (UMDNJ)-Robert Wood Johnson Medical School, 675 Hoes lane, Piscataway, NJ 08854, USA

[4]Department of Microbiology and Immunology, Drexel University College of Medicine, Philadelphia, PA 19129, USA.

[5]Department of Pathology, University of Pittsburgh, Pittsburgh, PA, 15261,USA

[6]Department of Medicinal Chemistry and Molecular Pharmacology, Purdue University, West Lafaytte, IN 47907, USA.

[7]Department of Chemistry, University of North Carolina at Chapel Hill, Chapel Hill, NC, 27599, USA,

[8]Department of Biochemistry and Biophysics, University of North Carolina at Chapel Hill, Chapel Hill, NC 27599, USA,

[9]The Lineberger Comprehensive Cancer Center, University of North Carolina at Chapel Hill, Chapel Hill, NC 27514, USA,

[10] Current address: Department of Pathology, University of Iowa Hospitals and Clinics, Iowa City, IA 52242, USA

Corresponding author: Sean Ekins, Ph.D., D.Sc., Collaborations in Chemistry, 601 Runnymede Avenue, Jenkintown, PA 19046. Phone 215-687-1320; Fax 215-481-0159;

* Email ekinssean@yahoo.com

Table S10. External Validation test Set Predictions for 4D-QSAR (R^2 = 0.02) (Bold = outliers).

Compound	Experimental Activity	Predicted Activity
ANDROSTANES		
Androstenedione	4.69	4.45
11-Ketoetiocholanone	4.39	4.67
Epitestosterone sulfate	5.47	4.55
5α-Androstane	2.00	5.73
4,16-Androstadien-3-one	5.15	3.74
PREGNANES		
Corticosterone	5.00	4.88
Cortisone	4.16	4.31
Pregnenolone	5.64	4.51
Pregnanediol glucuronide	4.26	5.07
17α,20β-Dihydroxyprogesterone	2.00	4.87
Dexamethasone	4.39	2.63
BILE ACIDS / SALTS		
Chenodeoxycholic acid	2.00	3.88
Glycodeoxycholic acid	2.00	5.93
Taurocholic acid	2.00	2.71
5β-cholestan-3α,7α,12α-triol	2.00	2.88
23-Nordeoxycholic acid	4.79	5.40
Petromyzonol sulfate	4.55	3.37
Scymnol – sulfated	4.31	2.97
Taurolithocholic acid 3-sulfate, disodium salt	4.08	4.77
α-Cholestanol	2.00	1.70

The following equations relate to the individual classes of steroids and show the cross validated correlations (XV-R^2). It would appear the larger bile salt/ acid dataset has the lowest cross validated correlation. These datasets were tested with external compounds although the data indicates a negative correlation for the androstanes (-0.77) and no correlation for the pregnanes and bile salts/ acids. In all the equations ACCEPTOR = hydrogen bond acceptor, ALL = any atom, NP = non-polar and POSITIVE = positively charged. It would seem overall to show mainly steric/non-polar interactions while hydrogen bond acceptor descriptors are highlighted in the androstanes equation.

Androstanes activity = 4.55 – 8.54 * GC1 (ACCEPTOR) –12.07 * GC2 (ALL) – 6.06 * GC3 (NP) + 1.52 * GC4 (NP)

N=20, R^2 = 0.94, Cross validated (XV)-R^2 = 0.89

Pregnanes activity = 3.21 – 7.12 * GC1 (NP) – 21.56 * GC2 (ALL) + 3.59 * GC3 (NP) -1.46 * GC4 (ALL) + 4.29 * GC5 (NP)

N=23, R^2 = 0.91, XV-R^2 = 0.84

Bile salts / acids activity = 0.43 – 23.81 * GC1 (ALL) + 34.3 * GC2 (ALL) – 6.07 * GC3 (ALL) + 5.88 * GC4 (ALL) – 5.04 * GC5 (ALL) + 3.89 * GC6 (NP) + 12.35 * GC7 (ALL) – 13.66 * GC8 (POSITIVE)

N=41, R^2 = 0.77, XV-R^2 = 0.64
