# Supplementary material for: Challenges Predicting Ligand-Receptor Interactions of Promiscuous Proteins: The Nuclear Receptor PXR
Source: PLoS Comput Biol. 2009 Dec 11;5(12):e1000594. doi: 10.1371/journal.pcbi.1000594 (PMC2781111; doi:10.1371/journal.pcbi.1000594)
Supplement: Table S11 — Experimental versus predicted pEC50 values for 115 compounds binding to PXR divided into four different substrate classes - 5D-QSAR. (0.03 MB PDF) [file pcbi.1000594.s011.pdf]

Challenges Predicting Ligand-Receptor Interactions of Promiscuous Proteins: The Nuclear Receptor PXR

Sean Ekins[1,2,3*], Sandhya Kortagere[4,] Manisha Iyer[5], Erica J. Reschly[5], Markus A. Lill[6], Matthew R. Redinbo[7,8,9] and Matthew D. Krasowski[5,10].

[1]Collaborations in Chemistry, 601 Runnymede Avenue, Jenkintown, PA 19046, USA

[2]Department of Pharmaceutical Sciences, University of Maryland, 20 Penn Street, Baltimore, MD 21201, USA

[3]Department of Pharmacology, University of Medicine & Dentistry of New Jersey (UMDNJ)-Robert Wood Johnson Medical School, 675 Hoes lane, Piscataway, NJ 08854, USA

[4]Department of Microbiology and Immunology, Drexel University College of Medicine, Philadelphia, PA 19129, USA.

[5]Department of Pathology, University of Pittsburgh, Pittsburgh, PA, 15261,USA

[6]Department of Medicinal Chemistry and Molecular Pharmacology, Purdue University, West Lafaytte, IN 47907, USA.

[7]Department of Chemistry, University of North Carolina at Chapel Hill, Chapel Hill, NC, 27599, USA,

[8]Department of Biochemistry and Biophysics, University of North Carolina at Chapel Hill, Chapel Hill, NC 27599, USA,

[9]The Lineberger Comprehensive Cancer Center, University of North Carolina at Chapel Hill, Chapel Hill, NC 27514, USA,

[10] Current address: Department of Pathology, University of Iowa Hospitals and Clinics, Iowa City, IA 52242, USA

Corresponding author: Sean Ekins, Ph.D., D.Sc., Collaborations in Chemistry, 601 Runnymede Avenue, Jenkintown, PA 19046. Phone 215-687-1320; Fax 215-481-0159;

* Email ekinssean@yahoo.com

Table S11. Experimental versus predicted pEC_{50} values for 115 compounds binding to PXR divided into four different substrate classes – 5D-QSAR
.

Compound name	Experimental EC_{50} (μM)	Predicted EC_{50} (μM)	Test set
ANDROSTANES			
17b-dihydroandrosterone	4.15	12.5	
Androstanedione	12.7	4.39	
Androstanol	6.27	7.87	
Androsterone	18.6	5.80	
Dihydrotestosterone	11.4	5.79	
Etiocholanolone	5.7	4.16	
**Androstenedione**	**20.6**	8.22	x
Testosterone	71.9	13.8	
DHEA	32.5	12.5	
Androstenol	5.5	9.28	
11b-Hydroxyetiocholanolone	19.2	8.71	
DHEA sulfate (sodium salt)	48.2	12.3	
Epiandrosterone	4.91	9.08	
**11-Ketoetiocholanone**	**41.1**	14.8	x
Epitestosterone	68	47.8	
Epitestosterone glucuronide	13.8	7.10	
**Epitestosterone sulfate**	**3.39**	7.53	x
Etiocholanolone glucuronide	10000	159	
**5a-Androstane**	**10000**	2.18	x
5a-Androstan-3b-ol	0.8	3.21	
16,(5a)-Androsten-3b-ol	4.77	6.31	
16,(5a)-Androsten-3-one	3	4.17	
5b-Androstan-3a-ol	1.41	3.16	
**4,16-Androstadien-3-one**	**7.1**	10.24	x
5,16-Androstadien-3b-ol	10000	16.32	
PREGNANES			
Pregnanediol	5.1	9.26	
Pregnanedione	2.6	2.49	
**Corticosterone**	**9.95**	9.85	x
Cortexolone	23	28.8	
Aldosterone	55	102	
**Cortisone**	**69.4**	93.3	x
Progesterone	14.7	5.50	
17-Hydroxyprogesterone	17.7	6.31	
Cortexone	2.44	7.93	
Cortisol	47.6	21.8	
17-Hydroxypregnenolone	33.5	13.6	
**Pregnenolone**	**2.3**	8.65	x

Pregnenolone carbonitrile (PCN)	10000	20.5	
Allopregnanolone	4.21	3.72	
Allopregnanediol	52.5	9.38	
**Pregnanediol glucuronide**	**55.1**	196	x
Cortol	47.2	43.4	
Cortolone	44.7	96.4	
Tetrahydrocortisone	52.7	65.8	
THDOC	12.5	38.7	
**17a,20b-Dihydroxyprogesterone**	**10000**	152	x
17a,20b-Dihydroxyprogesterone sodium sulfate	1.88	4.15	
5b-Pregnan-3a,20b,diol	3.81	2.97	
Tetrahydrocortisol	46.5	28.4	
Pregnanolone	10.4	6.38	
Pregnenolone sulfate	10000	37.2	
Levonorgestrol	4.30	11.1	
Norethindrone	25.60	74.7	
**Dexamethasone**	**41.00**	40.5	x
ESTRADIENES			
Estradiol	16	4.05	
Estrone	37.9	7.82	
Estriol	10000	23.3	
16a-Hydroxyestrone	2.5	11.0	
4-Methoxyestrone	4	11.6	
Estetrol	2.14	3.13	
2-Hydroxyestrone	3.6	11.8	
Estrone 3-sulfate	3.4	4.17	
Estradiol glucuronide	10000	42.1	
Estradiol 3-sulfate	0.89	1.918	
Ethinyl estradiol	1.9	6.33	
BILE ACIDS			
5b-Cholan-3a,7a,12a,24-tetrol (5b-Petromyzonol)	10000	337	
Hyodeoxycholic acid	38.1	32.5	
Taurohyodeoxycholic acid	19	19.3	
Murocholic acid (Murideoxycholic acid)	10000	26.1	
**Chenodeoxycholic acid**	**10000**	15.1	x
Glycochenodeoxycholic acid	10000	80.6	
Taurochenodeoxycholic acid	104	148	
Deoxycholic acid	50.2	31.2	
**Glycodeoxycholic acid**	**10000**	88.4	x
Taurodeoxycholic acid	10000	150	
Lithocholic acid	10	6.22	
Glycolithocholic acid	16.1	7.60	
**Taurolithocholic acid**	**19.8**	8.20	x
7,12-Diketolithocholic acid	35.5	36.6	
7-Ketolithocholic acid	21.5	17.9	

12-Ketolithocholic acid	31.3	12.6	
w-Muricholic acid	10000	117	
a-Muricholic acid	56	65.0	
b-Muricholic acid	10000	76.0	
Cholic acid	11.6	35.9	
Glycocholic acid	10000	124	
Taurocholic acid	10000	737	
**5b-cholestan-3a,7a,12a-triol**	**10000**	471	x
20a-hydroxycholesterol	10000	394	
Cholesterol	10000	146	
**23-Nordeoxycholic acid**	**16.4**	12.3	x
23-Norcholic acid	96	29.9	
3-Ketopetromyzonol	10000	255	
Petromyzonol	10000	180	
**Petromyzonol sulfate**	**28.1**	47.1	x
Allocholic acid	10000	28.6	
7,12-Dihydroxy-5-cholan-3-one-24-sulfate (3-Ketopetromyzonol sulfate)	10000	54.5	
3-Keto-7a,12a-dihydroxy-5a-cholanic acid	12.1	32.0	
**Scymnol - sulfated**	**49.3**	194	x
Scymnol - desulfated	10000	8814	
5a-cyprinol - sulfated	25.9	19.2	
5a-cyprinol - desulfated	10000	754	
Alligator bile acid	10000	398	
Myxinol - sulfated	10000	835	
Myxinol - desulfated	10000	1727	
Lithocholic acid 3-sulfate	117.9	21.0	
7-Ketodeoxycholic acid	58.2	74.0	
Glycolithocholic acid 3-sulfate, disodium salt	56.2	53.5	
**Taurolithocholic acid 3-sulfate, disodium salt**	**83.2**	63.8	x
a-Cholestanol	10000	92.3	
Tauro-b-muricholic acid	10000	917	
7a-Hydroxycholesterol	10000	219	
**3-Ketolithocholic acid**	**8.3**	9.44	x
Lithocholic acid acetate	1.2	1.42	
Lithocholic acid acetate methyl ester	1.1	4.24	
