# Supplementary material for: Challenges Predicting Ligand-Receptor Interactions of Promiscuous Proteins: The Nuclear Receptor PXR
Source: PLoS Comput Biol. 2009 Dec 11;5(12):e1000594. doi: 10.1371/journal.pcbi.1000594 (PMC2781111; doi:10.1371/journal.pcbi.1000594)
Supplement: Text S1 — In silico methodology: 3D-QSAR - CoMFA, CoMSIA, In silico methodology: 3D-QSAR - Catalyst, In silico methodology: 4D-QSAR, Supplemental results: CoMFA, CoMSIA and Catalyst. Supplemental data - pharmacophores output files from Discovery Studio Catalyst. (0.08 MB PDF) [file pcbi.1000594.s012.pdf]

Challenges Predicting Ligand-Receptor Interactions of Promiscuous Proteins: The Nuclear Receptor PXR

Sean Ekins[1,2,3*], Sandhya Kortagere[4,] Manisha Iyer[5], Erica J. Reschly[5], Markus A. Lill[6], Matthew R. Redinbo[7,8,9] and Matthew D. Krasowski[5,10].

[1]Collaborations in Chemistry, 601 Runnymede Avenue, Jenkintown, PA 19046, USA

[2]Department of Pharmaceutical Sciences, University of Maryland, 20 Penn Street, Baltimore, MD 21201, USA

[3]Department of Pharmacology, University of Medicine & Dentistry of New Jersey (UMDNJ)-Robert Wood Johnson Medical School, 675 Hoes lane, Piscataway, NJ 08854, USA

[4]Department of Microbiology and Immunology, Drexel University College of Medicine, Philadelphia, PA 19129, USA.

[5]Department of Pathology, University of Pittsburgh, Pittsburgh, PA, 15261,USA

[6]Department of Medicinal Chemistry and Molecular Pharmacology, Purdue University, West Lafaytte, IN 47907, USA.

[7]Department of Chemistry, University of North Carolina at Chapel Hill, Chapel Hill, NC, 27599, USA,

[8]Department of Biochemistry and Biophysics, University of North Carolina at Chapel Hill, Chapel Hill, NC 27599, USA,

[9]The Lineberger Comprehensive Cancer Center, University of North Carolina at Chapel Hill, Chapel Hill, NC 27514, USA,

[10] Current address: Department of Pathology, University of Iowa Hospitals and Clinics, Iowa City, IA 52242, USA

Corresponding author: Sean Ekins, Ph.D., D.Sc., Collaborations in Chemistry, 601 Runnymede Avenue, Jenkintown, PA 19046. Phone 215-687-1320; Fax 215-481-0159;

* Email ekinssean@yahoo.com

Text S1

***In silico* methodology: 3D-QSAR - CoMFA, CoMSIA**

3D-QSAR (CoMFA and CoMSIA) and 4D-QSAR molecular modeling calculations and visualizations outlined here were carried out on a Silicon Graphics Fuel workstation under the IRIX 6.5 operating system. The data set of 95 training and 20 test compounds used to carry out 4D-QSAR, CoMFA and CoMSIA are listed in Supplemental Table 1 along with their activity (-log EC_{50}) values which were used as the dependent variable. The modeling techniques were applied to the entire training set as well as to the three individual subsets of androstanes, pregnanes and bile acids/salts. The subset of estratrienes was determined to be too small to perform a reliable *in silico* analysis.

The CoMFA and CoMSIA methodologies have been described extensively and reviewed previously [30,36]. The 3D chemical structures initially constructed using HyperChem were imported into the SYBYL 7.1 software package (Tripos Associates, St. Louis, MO) and subsequent CoMFA and CoMSIA analyses were carried out for both the master training set of 95 compounds and the three subsets. Energy minimizations were performed using the Tripos force field [1] with a distance-dependent dielectric and the Powell conjugate gradient algorithm with a convergence criterion of 0.01 kcal/(mol A). Partial atomic charges were calculated for all compounds using the Gasteiger-Hückel method.

Multiple approaches to alignment were attempted. First, alignment of the training sets was carried out using the “align database” option in SYBYL 7.1 Due to the lack of reliable binding data between human PXR and the steroidal compounds in the data set, as

well as the large number of compounds being analyzed, the best alignment option was not immediately apparent to us. Common substructure alignment with an inertial grid orientation was attempted for the training sets using different template molecules. The final alignments were picked based on the quality and plausibility of the actual alignment as well as the statistical quality of the QSAR model derived from it. The best alignments of the master training set (N = 95), the subsets of pregnanes (N = 23) and bile acids /salts (N = 41) were achieved using the conformation of pregnanedione (compound # 27, Supplemental Table 1) as the template molecule. The androstane subset was best aligned using androstanol (compound #20, Supplemental Table 1) as the template molecule.

For CoMFA, all the molecules were placed in a 3D lattice with regular grid points separated by 2 Å. The van der Waals potentials and the Coulombic term representing the steric and electrostatic fields were calculated using the standard Tripos force field for CoMFA. A C_{sp3} atom with a formal charge of +1 and a van der Waals radius of 1.52 Å served as probe atom to generate steric (Lennard-Jones 6-12 potential) and electrostatic (Coulombic potential) field energies, which were obtained by summing the individual interaction energies between each atom of the molecule and the probe atom at every grid point. A distance-dependent dielectric constant was used. The steric and electrostatic fields were truncated at ± 30.00 kcal/mol.

A similar approach was used for CoMSIA as the aligned molecules were placed in a 3D lattice with regular grid points separated by 2 Å. The five physicochemical properties for CoMSIA (steric, electrostatic, hydrophobic, hydrogen-bond donor and acceptor) were evaluated using a common probe atom with 1 Å radius, +1.0 charge, and hydrophobic and hydrogen-bond property values of +1. The attenuation factor α, which

determines the steepness of the Gaussian function, was assigned a default value of 0.3 (39). The PLS technique was employed to generate a linear relationship that correlates changes in the various computed potential fields with changes in the corresponding experimental values of activities (-log IC_{50}) for the data set. Employing the CoMFA and CoMSIA potential energy fields for each molecule as the independent variable and the corresponding activity values as the dependent variable, PLS converts these descriptors to the so-called latent variables or principal components (PCs) consisting of linear combinations of the original independent variables.

To assess the internal predictive ability of the CoMFA and CoMSIA models, the 'leave-one-out' (LOO) cross-validation procedures was employed. Cross-validation determines the optimum number of PCs, corresponding to the smallest error of prediction and the highest q^2. PLS analysis was repeated without validation using the optimum number of PCs to generate final CoMFA and CoMSIA models from which the conventional correlation coefficient r^2 was derived. The utility of the 3D-QSAR models were determined by predicting the activities of the test set compounds that were not included in the training sets after aligning in the same way as those in the training set.

In silico methodology: 3D-QSAR - Catalyst

The pharmacophore modeling studies were carried out using Catalyst in Discovery Studio version 1.7 (Accelrys, San Diego, CA) running on a Sony Vaio laptop computer (Intel Pentium M processor). This methodology has been previously described [2]. Molecules were imported as an sdf file and the 3-D molecular structures were produced using up to 255 conformers with the best conformer generation method, allowing a maximum energy difference of 20 kcal/mol. Ten hypotheses were generated

using these conformers for each of the molecules and the EC_{50} values, after selection of the following features: hydrophobic, hydrogen bond acceptor, hydrogen bond donor and ring aromatic features. In addition, hypotheses were generated with up to 2 excluded volumes, variable weight and tolerances and a combination of excluded volumes and variable weight and tolerances. In all cases, after assessing all ten generated hypotheses, the one with lowest energy cost was selected for further analysis as this usually possessed features representative of all the hypotheses. The quality of the structure activity correlation between the estimated and observed activity values was estimated by means of an *r* value.

As Catalyst is commonly used with relatively small training sets (greater or equal to 16 molecules) we generated individual models for the different types of steroids only.

In silico **methodology: 4D-QSAR**

The 4D-QSAR methodology has been presented previously in detail [3]. Briefly, the commercial version (V3.0) of the 4D-QSAR package was employed in this study (4D-QSAR, Version 3.0; The Chem21 Group, Inc., Lake Forest, IL). This study uses a receptor-independent (RI-4D-QSAR) analysis. The first step in the analysis is to generate a reference grid cell lattice in which to place the 3D structure of each training set compound. This grid cell lattice is composed of a set of one angstrom cubes. The 3D structures of the training set compounds were then constructed and optimized in Hyperchem (Release 7.51 for Windows; Hypercube, Inc. Gainesville, FL) The preferred compound geometry was determined via molecular mechanics with an MM+ force field, and the partial charges were assigned using a semiempirical AM1 method implemented in the Hyperchem program [3].

The interaction pharmacophore elements, or IPEs were assigned to the intramolecular energy minimized 3D structure of each compound and the *conformational ensemble profile*, or CEP, was then generated for each training set compound. The seven IPEs used in 4D-QSAR analyses represent any/all atoms, non-polar atoms, polar positive atoms, polar negative atoms, hydrogen-bond acceptor atoms, hydrogen-bond donor atoms, aromatic atoms and non-hydrogen atoms. A molecular dynamics simulation (MDS) was used to create the CEP. The MOLSIM V3.0 (C. Doherty and The Chem21 Group, Inc., Lake Forest, IL) software package with the extended MM2 force field was utilized to perform the MDS. The molecular dielectric was set to 3.5, and the simulation temperature was fixed at 300 K. A sampling time of 100 ps was employed, over which a total of 1000 conformations of each compound were recorded. The CEP was created by recording the atomic coordinates and conformational energy every 0.1 ps throughout the simulation, resulting in 1000 "snapshots" of each compound as it traverses through the set of thermodynamically available conformer states.

Following generation of the CEP of each compound, the molecular alignments were chosen for the training set. Three-ordered atom alignment rules were used in this study. In general, the alignments are chosen to span the common framework (core) of the molecules in the training set so that information relating to the substituent properties of the compounds is obtained from the resulting models. This alignment strategy is reflected in those which were chosen and listed along with the steroidal core structure in Supplemental Table 7.

All conformations from the CEP of every compound are placed in the grid cell lattice space according to a selected trial alignment. The occupancy of the grid cells by

each IPE type is recorded over the CEP which then forms the set of grid cell occupancy descriptors, or GCODs which are utilized as the pool of trial descriptors in the model building and optimization process. The genetic function approximation (GFA) is used to optimize the 4D-QSAR models [4].

Since GFA typically generates a family of possible models, the best models in the 4D-QSAR study were chosen based on a number of different criteria. In addition to the leave-one-out cross-validated correlation coefficient, or q^2, other statistical measures such as r^2, standard error (SE), and lack-of-fit (LOF) were considered as indicators of model fitness [4]. The optimal number of descriptor terms to include in the best model was determined by plotting the number of model terms versus the cross-validated correlation coefficient (data not shown). The point of the plot at which the q^2 did not significantly increase with the addition of an additional model term was chosen as the optimal number of model terms. Test sets not included in the training sets were also used to evaluate the predictive power of the 4D-QSAR models.

The active conformation of each of the compounds in the training sets was postulated relative to the best 4D-QSAR model for the respective set. This was accomplished by first determining the conformations of the CEP that are within a threshold energy limit of 5 kcal, i.e., only thermodynamically accessible conformations are considered, and then determining which of these possible conformations has the highest activity as predicted by the model.

Supplemental results: CoMFA, CoMSIA and Catalyst

Each of the individual classes of steroids were used to create individual models with improved R^2 values. For androstanes (Training Set: N=20; Test Set: N=5) after PLS region focusing outlier removal XV-R^2 = 0.57, and R^2 = 0.96 (6 component PLS model; 43% Steric, 57% Electrostatic). 5α-Androstan-3β-ol (pIC_{50} = 6.1) is shown with the steric component of the CoMFA model in Figure S1A. 5α-Androstan-3β-ol shown with the steric component of the CoMFA model is shown in Figure S1B. A CoMSIA model was also created for androstanes. Using the PLS focused region, CoMSIA components were calculated. After omitting the same two outliers as in the CoMFA model, the best CoMSIA model for the Androstanes subset has an XV-R^2 = 0.62, and R^2 = 0.95 (7 component PLS model; 23% Steric, 24% Hydrophobic and 53% hydrogen bond acceptor). 17β-dihydroandrosterone (pIC_{50} = 5.38) is shown with the steric component of the CoMSIA model (Figure S2A), with the hydrophobic component of the CoMSIA model (Figure S2B), and with the hydrogen bond acceptor component of the CoMSIA model (Figure S2C).

For pregnanes (Training set: N=23; Test set: N=6) (Table S6), the table lists the activities of the training set as predicted by leave-one-out cross validation. The best model had XV-R^2 = 0.80 and R^2 = 0.92 (4 component model; 50% Steric, 50% Electrostatic). Pregnanedione (pIC_{50} = 5.59) is shown with the steric component of the CoMFA model (Figure S3A) and with the electrostatic component of the CoMFA model (Figure S3B). A CoMSIA model was also created for pregnanes. Using the PLS focused region, CoMSIA components were calculated. Omitting the same outlier as in the CoMFA model, the best CoMSIA model for the Pregnanes subset has an XV-R^2 = 0.74, and R^2 = 0.96 (5 component PLS model; 20% Steric, 48% Hydrophobic and 32%

electrostatic).The inactive training set molecule Pregnenolone Carbonitrile (PCN) (pIC_{50} = 2.00) is shown with the steric component of the CoMSIA model (Figure S4A), with the electrostatic component of the CoMSIA model and with the hydrophobic component of the CoMSIA model (Figure S4C).

For bile acids / bile salts (Training Set: N=41; Test Set: N=9), the best CoMFA model was XV-R^2 = 0.64 and R^2 = 0.97 (6 component PLS model; 34% Steric, 66% Electrostatic). Lithocholic acid acetate (pIC_{50} = 5.92) is shown with the steric component of the CoMFA model (Figure S5A), with the electrostatic component of the CoMFA model (Figure S5B). A CoMSIA model was created for bile acids and salts. The best CoMSIA model for the Bile Acids /Salts subset has an XV-R^2 = 0.63, and R^2 = 0.98 (7 component PLS model; 49% Electrostatic, 21% Hydrophobic and 30% hydrogen bond donor). Hyodeoxycholic acid (pIC_{50} =4.42) is shown with electrostatic components of the CoMSIA model (Figure S6A), with the hydrophobic component (Figure S6B) and with the hydrogen bond donor component (Figure S6C).

We attempted to produce CoMFA models for the entire set (Training set, n=95). The best CoMFA model (9 component partial least squares (PLS) model; 51% steric, 49% electronic) had R^2 = 0.90 but XV-R^2 = 0.52. The predictions for the external test set (n=20) did not result in a significant correlation. Similar results were obtained for the steroidal compound subsets.

Using the pharmacophore approach for the individual steroids, the training set r values were quite low (0.62-0.84) without excluded volumes, increasing with excluded volumes (0.75-0.86), variable weight and tolerances (0.71–0.84) and excluded volumes with variable weight and tolerances (0.81-0.93) (Table S8). Three out of four

pharmacophores showed an improvement in r statistics with the addition of 2 excluded volumes while all pharmacophores had improved r values with both excluded volumes and variable weights and tolerances. All PXR pharmacophores (Figure S8) had at least 2 hydrophobes and a hydrogen bond acceptor in common.

The bile acid pharmacophore (Figure S8A) had the largest cost difference [5] out of all the pharmacophores (nearly 100 units, Supplemental data) suggesting this is likely to be the most significant, as the cost difference is frequently used as a measure of model quality [6]. The combination of variable weights and tolerances as well as excluded volumes in all cases narrowed or removed the total cost to null cost difference. This is indicative of less statistically relevant hypotheses even though the r values generally were the highest. The estratriene (Figure S8B) and pregnane pharmacophores (Figure S8D) had the most features (6) while the androstane pharmacophore (Figure S8C) had the least features (3).

Supplemental References

1. Clark MA, Cramer RD, van Op den Bosch N (1989) Validation of the general purpose Tripos 5.2 force field. J Comput Chem 10: 982-1012.
2. Ekins S, Chang C, Mani S, Krasowski MD, Reschly EJ, et al. (2007) Human pregnane X receptor antagonists and agonists define molecular requirements for different binding sites. Mol Pharmacol 72: 592-603.
3. Hopfinger AJ, Wang S, Tokarski JS, Jin B, Albuquerque M, et al. (1997) Construction of 3D-QSAR models using the 4D-QSAR analysis formalism. . J Am Chem Soc 119: 10509-10524.

4. Rogers D, Hopfinger AJ (1994) Application of genetic function approximation to quantitative structure-activity relationships and quantitative structure-property relationships. J Chem Inf Comp Sci 34: 854-866.

5. Guner OF, editor (2000) Pharmacophore, perception, development, and use in drug design. San Diego: University International Line.

6. Li H, Sutter J, Hoffman R (2000) HypoGen: An automated system for generating 3D predictive pharmacophore models. In: Guner OF, editor. Pharmacophore perception, development, and use in drug design. San Diego: IUL. pp. 173-189.

7. Khandelwal A, Krasowski MD, Reschly EJ, Sinz MW, Swaan PW, et al. (2008) Machine learning methods and docking for predicting human pregnane X receptor activation. Chem Res Toxicol 21: 1457-1467.

Supplemental data - pharmacophores

Supplemental data – Bile acids hypothesis

```
Definition:      HBA       HBA     HYDROPHOBIC   HYDROPHOBIC    2
EVolumes

Weights:       3.32407     3.32407     3.32407     3.32407
Tolerances:   1.60   2.20   1.60   2.20     1.60        1.60
Coords: X     -3.15   -6.05  2.81    2.71     0.34        -0.78
      : Y     5.13    4.48   -6.18   -8.77     3.14        -2.10
      : Z     -2.98   -3.41  2.36    3.92      -3.14       2.24
              o--------->   o--------->      o          o
Excluded volume ( 2.90 , 1.82 , -4.25 ) Tol: 120.00
Excluded volume ( -3.52 , 2.80 , -4.79 ) Tol: 120.00
HBA        o
       --->  3.0
HBA        o 13.9   15.0
       --->  16.6   17.5   3.0
HYDROPHOBIC  o 4.0    6.5    11.1    14.1
```

HYDROPHOBIC o 9.2 10.2 5.4 7.7 7.6

Maximum Fit: 13.2963

Name	Fit	Cnf/Enan	Mapping	Est	Act	Error	Unc
BI049	12.21	120	+ [26 25 21 6]	6.1	1	+ 6.1	3
BI050	13.00	107	+ [26 25 21 6]	0.99	1	- 1	3
BI048	12.37	35	+ [25 65 21 7]	4.2	8	- 1.9	3
BI011	11.34	49	+ [26 25 21 7]	46	10	+ 4.6	3
BI020	11.54	70	+ [27 25 21 19]	28	11	+ 2.6	3
BI033	11.86	19	+ [66 65 21 19]	14	12	+ 1.1	3
BI012	10.77	9	+ [26 25 21 19]	170	16	+ 11	3
BI026	11.05	12	+ [64 23 21 19]	89	16	+ 5.5	3
BI003	11.27	65	+ [66 25 21 19]	53	19	+ 2.8	3
BI013	10.45	124	+ [26 25 21 6]	350	19	+ 18	3
BI015	9.91	48	+ [26 * 21 19]	1200	21	+ 58	3
BI036	9.73	115	+ [72 * 21 19]	1800	25	+ 73	3
BI030	9.78	63	+ [72 * 21 19]	1700	28	+ 59	3
BI016	10.85	42	+ [27 25 21 19]	140	31	+ 4.6	3
BI014	9.76	16	+ [27 * 21 19]	1700	35	+ 49	3
BI002	10.67	82	+ [27 25 21 19]	210	38	+ 5.6	3
BI034	11.97	55	+ [83 33 21 19]	11	49	- 4.6	3
BI008	11.10	58	+ [26 25 21 6]	80	50	+ 1.6	3
BI018	10.42	8	+ [27 25 21 19]	380	56	+ 6.7	3
BI043	11.01	144	+ [26 25 21 6]	97	56	+ 1.7	3
BI042	10.62	48	+ [26 25 21 19]	240	58	+ 4.1	3
BI044	11.50	151	+ [78 67 2 15]	31	83	- 2.6	3
BI027	11.23	18	+ [65 23 21 19]	59	96	- 1.6	3
BI007	9.83	101	+ [* 27 19 21]	1500	100	+ 14	3
BI041	11.09	62	+ [26 70 21 2]	81	120	- 1.4	3
BI024	8.53	19	+ [* 64 27 13]	29000	10000	+ 2.9	3
BI006	9.74	10	+ [* 25 21 19]	1800	10000	- 5.5	3
BI025	9.14	9	+ [* 65 25 6]	7100	10000	- 1.4	3
BI023	9.73	17	+ [* 33 21 19]	1800	10000	- 5.4	3
BI005	9.65	31	+ [27 25 21 19]	2200	10000	- 4.5	3
BI038	9.67	54	+ [* 33 21 19]	2100	10000	- 4.7	3
BI004	9.81	20	+ [* 25 21 19]	1500	10000	- 6.5	3
BI019	9.76	21	+ [27 * 21 19]	1700	10000	- 5.8	3
BI047	8.95	19	+ [* 65 25 13]	11000	10000	+ 1.1	3
BI046	9.69	47	+ [* 25 21 19]	2000	10000	- 4.9	3
BI040	9.28	99	+ [74 * 21 26]	5300	10000	- 1.9	3
BI039	10.46	124	+ [74 83 21 26]	340	10000	- 29	3
BI010	9.81	110	+ [* 25 21 6]	1600	10000	- 6.4	3
BI021	9.72	66	+ [* 25 21 19]	1900	10000	- 5.3	3
BI045	8.86	31	+ [* 65 21 6]	14000	10000	+ 1.4	3
BI032	9.46	47	+ [68 * 21 19]	3400	10000	- 2.9	3

```
BI031  9.87 24    + [27  *   21  19  ]   1300      10000     -   7.4     3
BI035  9.77 126   + [*   33  21  19  ]   1700      10000     -   5.9     3
BI009 10.37 66    + [26  25  21  6   ]    420      10000     -  24       3
BI029  9.65 42    + [63  *   21  19  ]   2200      10000     -   4.5     3
BI037  9.68 18    + [*   33  21  19  ]   2100      10000     -   4.8     3
BI001  9.62 16    + [*   30  21  19  ]   2400      10000     -   4.2     3
BI017  9.70 80    + [27  *   21  19  ]   2000      10000     -   5.1     3
BI028  9.44 18    + [60  68  21  19  ]   3600      10000     -   2.8     3
BI022  9.72 151   + [*   27  19  21  ]   1900      10000     -   5.3     3
 totalcost=272.471 RMS=1.74875 correl=0.79476
 Cost components: Error=244.633 Weight=10.7362 Config=17.1017 Tolerance=0
```

Fixed Cost:
 totalcost=186.407 RMS=0 correl=0
 Cost components: Error=168.18 Weight=1.12499 Config=17.1017 Tolerance=0

estratrienes

```
Definition:        HBA          HBD       HYDROPHOBIC    HYDROPHOBIC      2
EVolumes

Weights:        2.88224       2.88224       2.88224       2.88224
Tolerances:    1.60    2.20   1.60    2.20      1.60          1.60
Coords: X      -4.09   -4.74  5.89    7.62      0.80          -1.44
      : Y      -1.44   -4.19  -0.46   -2.89     1.84          -0.24
      : Z      -1.66   -2.66  -0.47   0.04      -0.14         1.30
               o--------->    o--------->       o             o
Excluded volume ( 7.30 , 4.80 , -1.39 ) Tol: 120.00
Excluded volume ( -6.45 , -2.66 , -2.36 ) Tol: 120.00
HBA          o
       --->  3.0
HBD          o 10.1    11.5
       --->  11.9    12.7   3.0
HYDROPHOBIC  o 6.1     8.6    5.6     8.3
HYDROPHOBIC  o 4.2     6.5    7.5     9.5       3.4

Maximum Fit: 11.5289

Name  Fit  Cnf/Enan      Mapping        Est        Act      Error    Unc
ES10 10.33 23    + [20  26  6   11  ]      9         0.89  +  10       3
ES11 10.37 6     + [20  19  6   11  ]      8.3       1.9   +   4.4     3
ES6  10.01 7     + [21  19  6   11  ]     19         2.1   +   8.8     3
ES4  10.96 8     + [20  19  7   13  ]      2.1       2.5   -   1.2     3
ES8  10.56 27    + [20  23  6   12  ]      5.3       3.4   +   1.6     3
```

ES7	10.87	4	+ [20 19 7 12]	2.7	3.6	- 1.4	3
ES5	10.50	9	+ [20 19 6 12]	6.1	4	+ 1.5	3
ES1	9.50	7	+ [20 19 7 11]	61	16	+ 3.8	3
ES2	10.73	4	+ [20 19 6 12]	3.6	38	- 10	3
ES9	8.52	75	+ [* 30 2 7]	590	10000	- 17	3
ES3	8.40	4	+ [* 19 6 11]	780	10000	- 13	3

totalcost=66.9988 RMS=1.61387 correl=0.8647
Cost components: Error=51.3248 Weight=5.39203 Config=10.2819 Tolerance=0

Fixed Cost:
totalcost=48.4066 RMS=0 correl=0
Cost components: Error=36.9997 Weight=1.12499 Config=10.2819 Tolerance=0

Androstanes

```
Definition:      HBA        HYDROPHOBIC   HYDROPHOBIC
Weights:       2.89255      2.89255      2.89255
Tolerances:   1.60   2.20     1.60         1.60
Coords: X     16.54  18.38    12.60        12.84
      : Y      8.16   6.67     8.73        11.73
      : Z    -12.35 -14.19   -10.46       -10.88
               o--------->      o            o
HBA             o
          --->  3.0
HYDROPHOBIC   o 4.4    7.2
HYDROPHOBIC   o 5.3    8.2       3.0
```

Maximum Fit: 8.67764

Name	Fit	Cnf/Enan	Mapping	Est	Act	Error	Unc
AN20	8.35	1	+ [51 19 6]	12	0.8	+ 15	3
AN23	8.42	1	+ [50 6 19]	10	1.4	+ 7.4	3
AN22	8.27	1	+ [48 19 6]	15	3	+ 5	3
AN17	8.33	1	+ [51 15 12]	13	3.4	+ 3.8	3
AN1	8.33	1	+ [20 7 19]	13	4.2	+ 3.1	3
AN21	8.37	1	+ [48 19 6]	12	4.8	+ 2.5	3
AN13	8.36	1	+ [49 19 6]	12	4.9	+ 2.5	3
AN10	8.29	1	+ [48 7 19]	14	5.5	+ 2.6	3
AN6	8.37	1	+ [48 6 19]	12	5.7	+ 2.1	3
AN3	8.32	1	+ [51 6 19]	13	6.3	+ 2.1	3
AN24	8.18	1	+ [46 19 7]	18	7.1	+ 2.6	3
AN5	8.24	1	+ [20 19 6]	16	11	+ 1.4	3
AN2	8.27	1	+ [48 19 6]	15	13	+ 1.2	3
AN16	8.20	1	+ [55 16 18]	18	14	+ 1.3	3
AN4	8.32	1	+ [49 6 19]	13	19	- 1.4	3

```
AN11  8.33 1    + [47  6   19  ]    13        19    -   1.5    3
AN7   8.20 1    + [20  19  7   ]    18        21    -  1.2     3
AN9   8.22 1    + [46  19  6   ]    17        32    -  1.9     3
AN14  8.34 1    + [46  6   19  ]    13        41    -  3.3     3
AN12  7.59 1    + [50  2   19  ]    72        48    +  1.5     3
AN15  8.17 1    + [20  19  7   ]    19        68    -  3.6     3
AN8   8.18 1    + [20  19  7   ]    18        72    -  4       3
AN25  8.22 1    + [46  19  6   ]    17     10000    - 600      3
AN18  6.54 1    + [57  1   6   ]   800     10000    -  12      3
AN19  5.78 1    + [*   3   19  ]  4500     10000    -  2.2     3
 totalcost=127.707 RMS=1.58283 correl=0.747054
 Cost components: Error=115.407 Weight=5.49234 Config=6.80735 Tolerance=0
```

```
Fixed Cost:
 totalcost=92.0225 RMS=0 correl=0
 Cost components: Error=84.0901 Weight=1.12499 Config=6.80735 Tolerance=0
```

Pregnanes

```
Definition:        HBA        HYDROPHOBIC    HYDROPHOBIC    HYDROPHOBIC
            2 EVolumes

Weights:        2.94211        2.94211        2.94211        2.94211
Tolerances:    1.60    2.20       1.60          1.60          1.60
Coords: X      4.04    5.85       -2.46         1.72          -1.83
      : Y     0.21    0.84       1.36          0.00          -1.44
      : Z     -1.16   -3.47      -0.72         2.58          1.30
              o--------->        o             o             o
Excluded volume ( -4.41 , -4.33 , -5.64 ) Tol: 120.00
Excluded volume ( -0.39 , -5.29 , -4.75 ) Tol: 120.00
HBA         o
        --->  3.0
HYDROPHOBIC  o 6.6     8.8
HYDROPHOBIC  o 4.4     7.4       5.5
HYDROPHOBIC  o 6.6     9.3       3.5          4.0

Maximum Fit: 11.7684

Name  Fit  Cnf/Enan      Mapping        Est       Act      Error    Unc
PR22 10.56 18    + [31  19  18  7   ]    25        1.9   +  13       3
PR12 10.92 18    + [55  19  15  7   ]    11        2.3   +   4.7     3
PR9  11.21 22    + [28  19  15  7   ]    5.6       2.4   +   2.3     3
PR2  11.05 31    + [29  19  15  6   ]    8.1       2.6   +   3.1     3
PR23 10.98 5     + [22  15  19  18  ]    9.5       3.8   +   2.5     3
PR14 10.99 11    + [52  19  15  7   ]    9.3       4.2   +   2.2     3
```

PR27	10.87	14	+	[19	31	6	11]	12	4.3	+	2.8	3
PR1	10.95	11	+	[22	15	19	18]	10	5.1	+	2	3
PR3	11.22	16	+	[30	19	15	7]	5.4	10	-	1.8	3
PR25	10.45	4	+	[23	1	16	8]	32	10	+	3.1	3
PR20	10.50	12	+	[57	19	15	6]	29	13	+	2.3	3
PR7	10.93	11	+	[23	19	15	7]	11	15	-	1.4	3
PR8	9.83	12	+	[23	19	13	6]	130	18	+	7.5	3
PR4	10.32	6	+	[29	19	14	7]	43	23	+	1.9	3
PR28	10.47	6	+	[19	27	6	11]	31	26	+	1.2	3
PR11	10.28	16	+	[54	19	15	7]	48	34	+	1.4	3
PR29	10.66	24	+	[29	31	7	18]	20	41	-	2.1	3
PR18	10.78	31	+	[33	19	18	7]	15	45	-	3	3
PR24	10.14	3	+	[31	19	15	6]	65	46	+	1.4	3
PR17	10.50	19	+	[33	19	14	6]	29	47	-	1.6	3
PR10	10.44	86	+	[29	19	18	7]	33	48	-	1.5	3
PR15	10.29	5	+	[57	19	15	7]	46	52	-	1.1	3
PR19	10.22	8	+	[31	19	13	6]	55	53	+	1	3
PR5	10.70	10	+	[30	19	15	6]	18	55	-	3.1	3
PR16	10.93	167	+	[26	2	13	6]	11	55	-	5.1	3
PR6	10.13	14	+	[29	19	13	6]	66	69	-	1	3
PR21	8.74	11	+	[22	11	6	19]	1700	10000	-	6	3
PR13	8.67	7	+	[54	7	11	19]	1900	10000	-	5.2	3
PR26	10.13	73	+	[57	15	2	11]	67	10000	-	150	3

totalcost=141.957 RMS=1.29101 correl=0.807141
Cost components: Error=121.712 Weight=5.99085 Config=14.2542 Tolerance=0

Fixed Cost:
totalcost=112.924 RMS=0 correl=0
Cost components: Error=97.5445 Weight=1.12499 Config=14.2542 Tolerance=0
