# Supplementary figures and images for: Challenges Predicting Ligand-Receptor Interactions of Promiscuous Proteins: The Nuclear Receptor PXR
Source: PLoS Comput Biol. 2009 Dec 11;5(12):e1000594. doi: 10.1371/journal.pcbi.1000594 (PMC2781111; doi:10.1371/journal.pcbi.1000594)

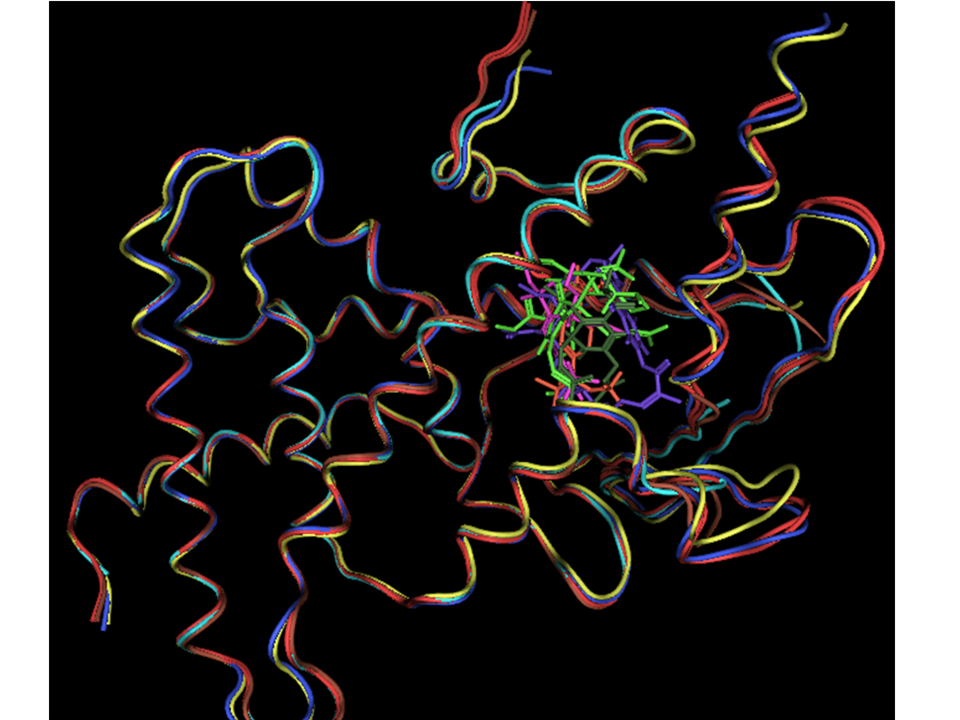

Supplement: Figure S1 — Structural superposition of six PXR crystal structures are shown in ribbon models and colored 1M13 (red), 1NRL (orange), 1SKX (cyan), 2O9I (blue), 2QNV (yellow) and PXR-EST (brown). The co-crystallized ligands are shown as sticks and colored blue for rifampicin, orange for colupulone, dark green for hyperforin, light green for N-{4-[2,2,2-trifluoro-1-hydroxy-1-(trifluromethyl)-ethyl]phenyl}benzenesulfonamide and pink for 17β-estradiol. (0.68 MB TIF) [file pcbi.1000594.s013.tif]

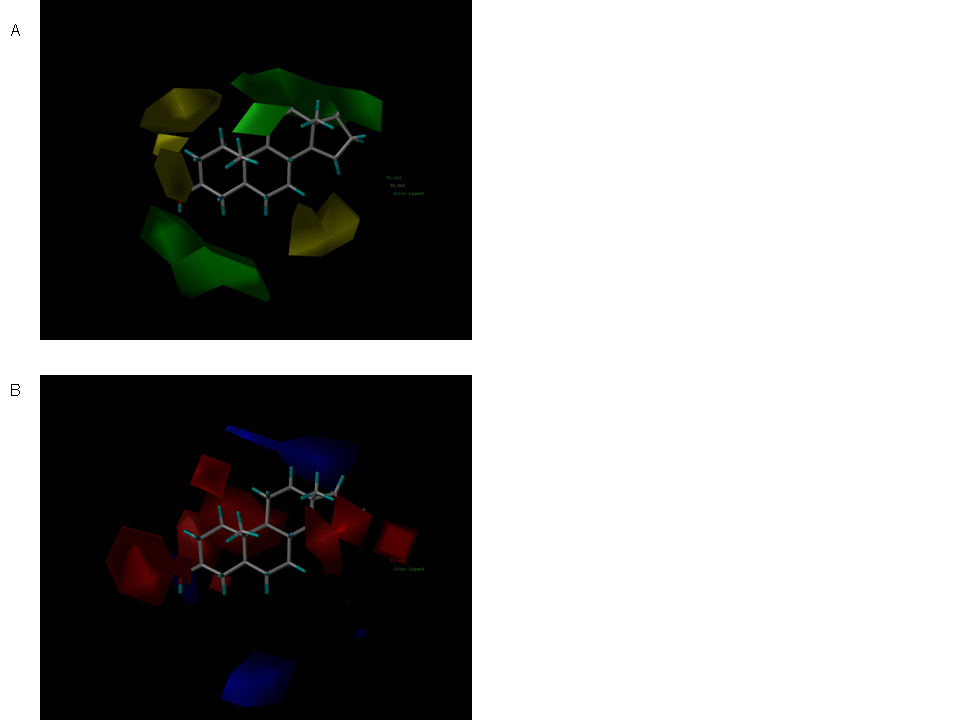

Supplement: Figure S2 — CoMFA models for androstanes. A 5α-Androstan-3β-ol (pIC50 = 6.1) shown with the steric component of the CoMFA model. Green denotes areas where steric bulk is favorable for bioactivity while yellow shows areas where steric bulk is not favored. B 5α-Androstan-3β-ol shown with the electrostatic component of the CoMFA model. Blue denotes areas where positive charge is favorable for bioactivity while red shows areas where negative charge is favored. (0.22 MB TIF) [file pcbi.1000594.s014.tif]

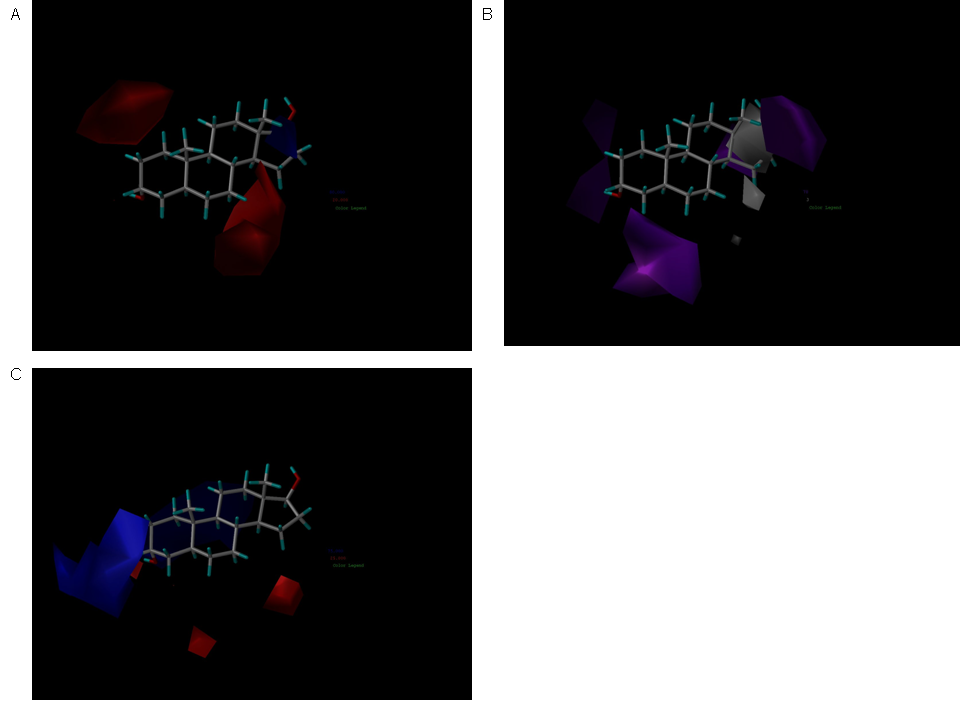

Supplement: Figure S3 — CoMSIA models for androstanes. A - 17β-dihydroandrosterone (pIC50 = 5.38) with the steric component of the CoMSIA model. Blue denotes areas where steric bulk is favorable for bioactivity while red shows areas where steric bulk is not favored. B 17β-dihydroandrosterone with the hydrophobic component of the CoMSIA model. Purple denotes areas where hydrophobic groups are favorable for bioactivity while grey shows areas where hydrophobic groups are not preferred. C 17β-dihydroandrosterone with the hydrogen bond acceptor component of the CoMSIA model. Blue denotes areas where acceptor groups are favorable for bioactivity while red shows areas where acceptor groups are not preferred. (0.24 MB TIF) [file pcbi.1000594.s015.tif]

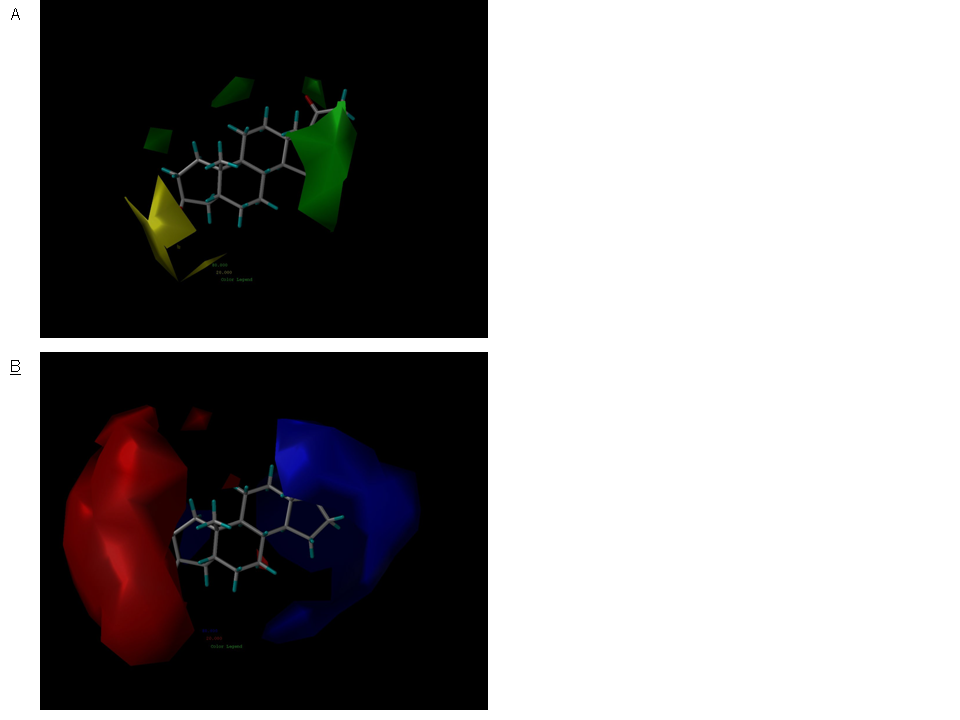

Supplement: Figure S4 — CoMFA models for pregnanes. A Pregnanedione (pIC50 = 5.59) shown with the steric component of the CoMFA model. Green denotes areas where steric bulk is favorable for bioactivity while yellow shows areas where steric bulk is not favored. B Pregnanedione shown with the electrostatic component of the CoMFA model. Blue denotes areas where positive charge is favorable for bioactivity while red shows areas where negative charge is favored. (0.26 MB TIF) [file pcbi.1000594.s016.tif]

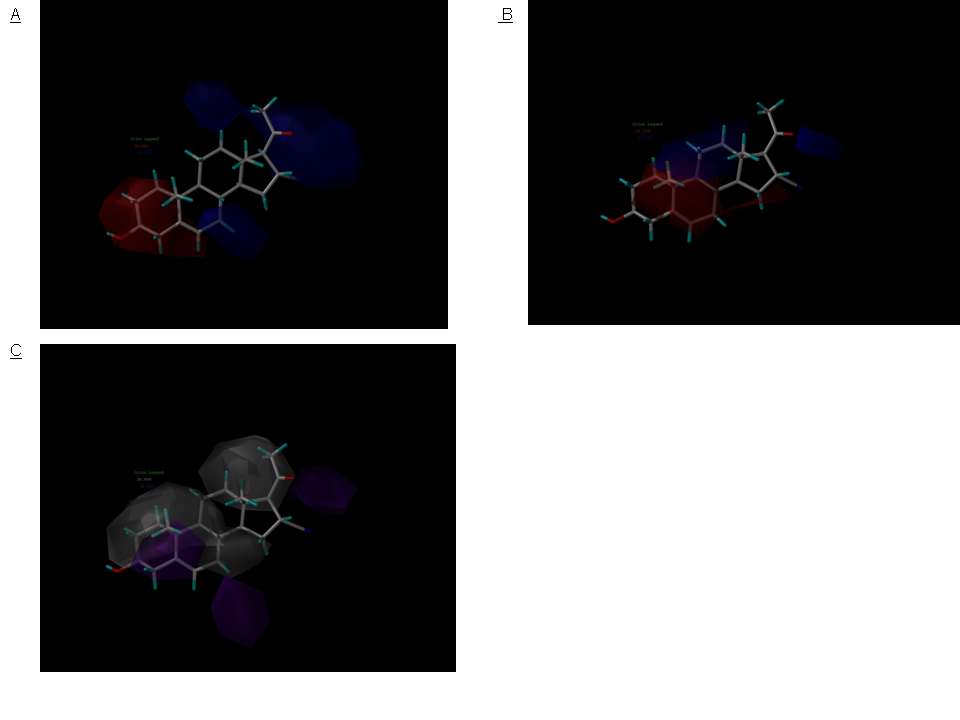

Supplement: Figure S5 — CoMSIA models for Pregnanes. A. Inactive training set molecule Pregnenolone Carbonitrile (PCN) (pIC50 = 2.00) with the steric component of the CoMSIA model. Blue denotes areas where steric bulk is favorable for bioactivity while red shows areas where steric bulk is not favored. B Inactive training set molecule PCN shown with the electrostatic component of the CoMSIA model. Blue denotes areas where positive charge is favorable for bioactivity while red shows areas where negative charge is favored. C. Inactive training set molecule PCN with the hydrophobic component of the CoMSIA model. Purple denotes areas where hydrophobic groups are favorable for bioactivity while grey shows areas where hydrophobic groups are not preferred. (0.23 MB TIF) [file pcbi.1000594.s017.tif]

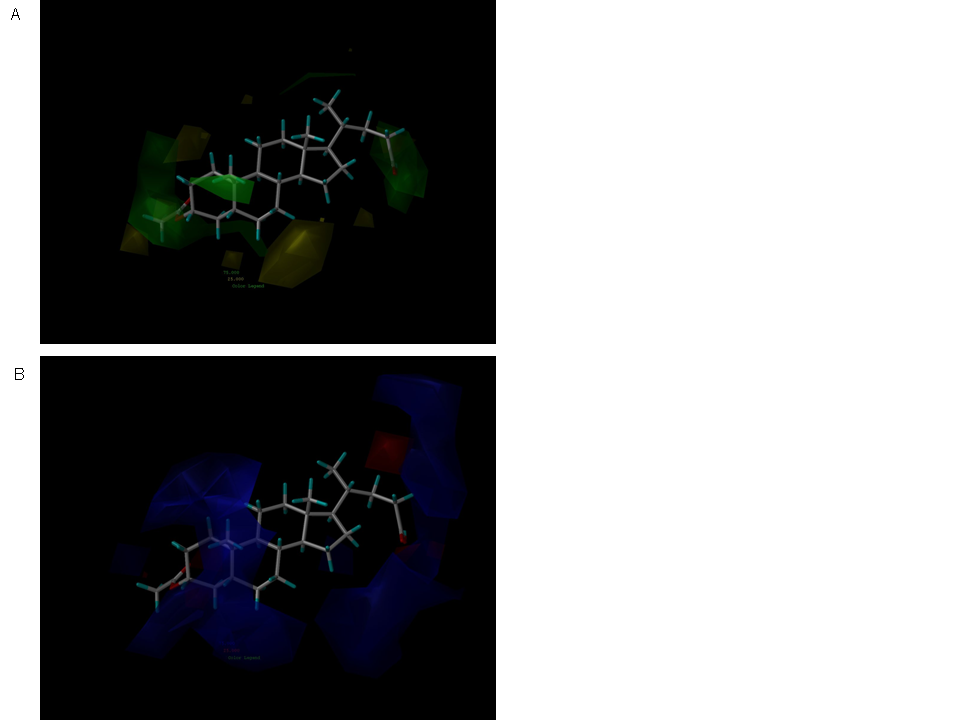

Supplement: Figure S6 — A. CoMFA models for bile acids and bile salts. Lithocholic acid acetate (pIC50 = 5.92) shown with the steric component of the CoMFA model. Green denotes areas where steric bulk is favorable for bioactivity while yellow shows areas where steric bulk is not favored. B Lithocholic acid acetate shown with the electrostatic component of the CoMFA model. Blue denotes areas where positive charge is favorable for bioactivity while red shows areas where negative charge is favored. (0.27 MB TIF) [file pcbi.1000594.s018.tif]

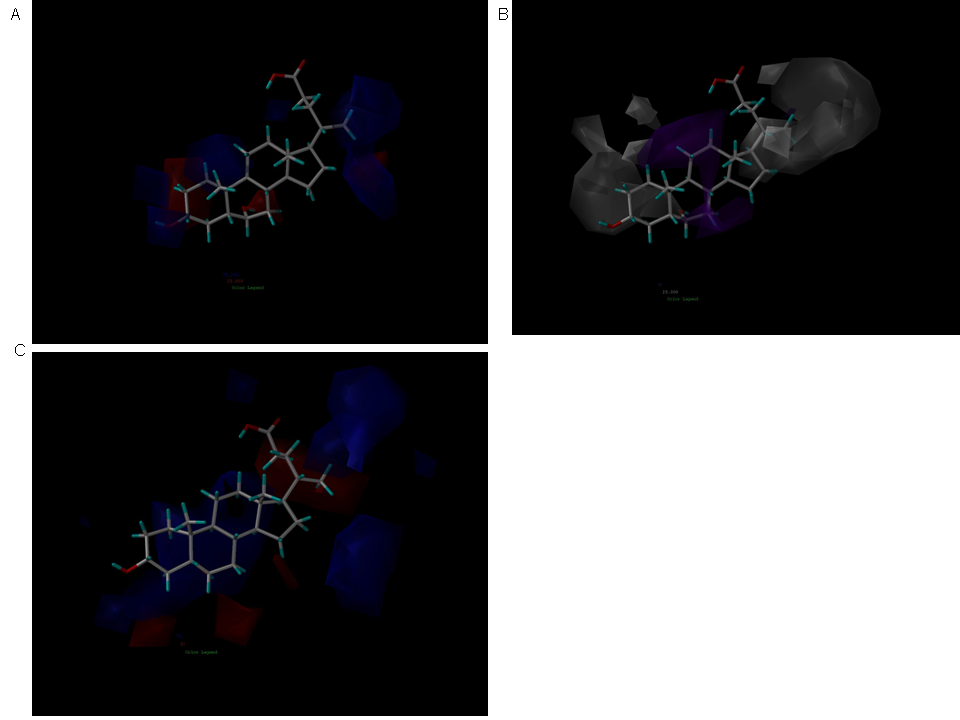

Supplement: Figure S7 — CoMSIA models of bile acids and bile salts. Using the PLS focused region, CoMSIA components were calculated. A. Hyodeoxycholic acid (pIC50 = 4.42) shown with electrostatic components of the CoMSIA model. Blue denotes areas where positive charge is favorable for bioactivity while red shows areas where negative charge is favored. B. Hyodeoxycholic acid with the hydrophobic component of the CoMSIA model. Purple denotes areas where hydrophobic groups are favorable for bioactivity while grey shows areas where hydrophobic groups are not preferred. C. Hyodeoxycholic acid with the hydrogen bond donor component of the CoMSIA model. Blue denotes areas where donor groups are favorable for bioactivity while red shows areas where donor groups are not preferred. (0.31 MB TIF) [file pcbi.1000594.s019.tif]

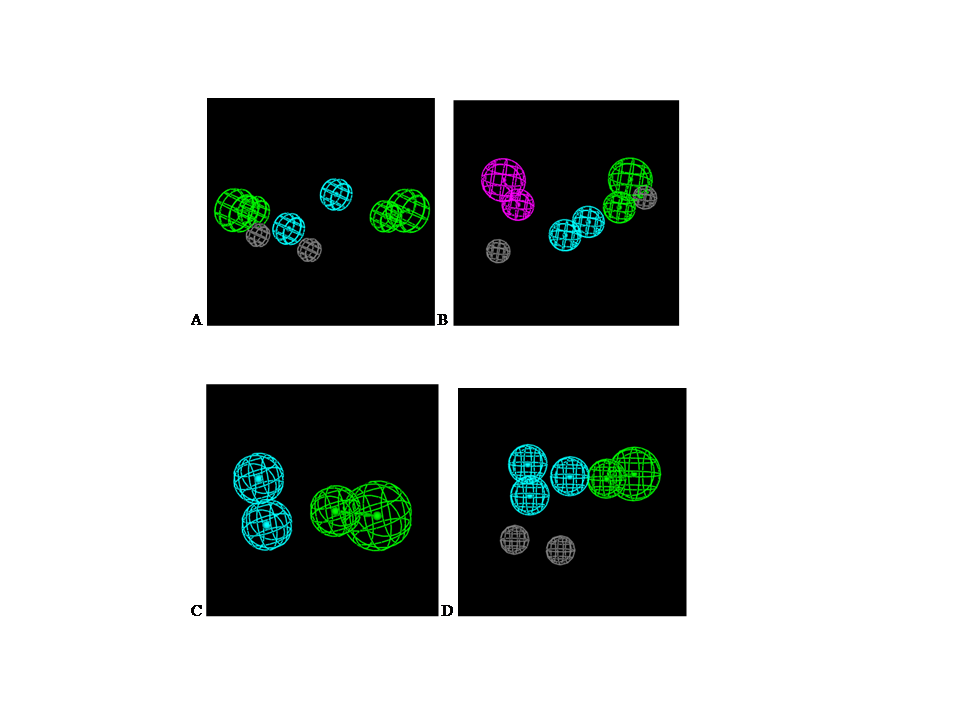

Supplement: Figure S8 — Catalyst PXR pharmacophores A. Bile acids, B. Estratrienes, C. Androstanes, D Pregnanes pharmacophore features represent Green = hydrogen bond acceptor, purple = hydrogen bond donor, blue = Hydrophobic, gray = excluded volumes. (0.15 MB TIF) [file pcbi.1000594.s020.tif]

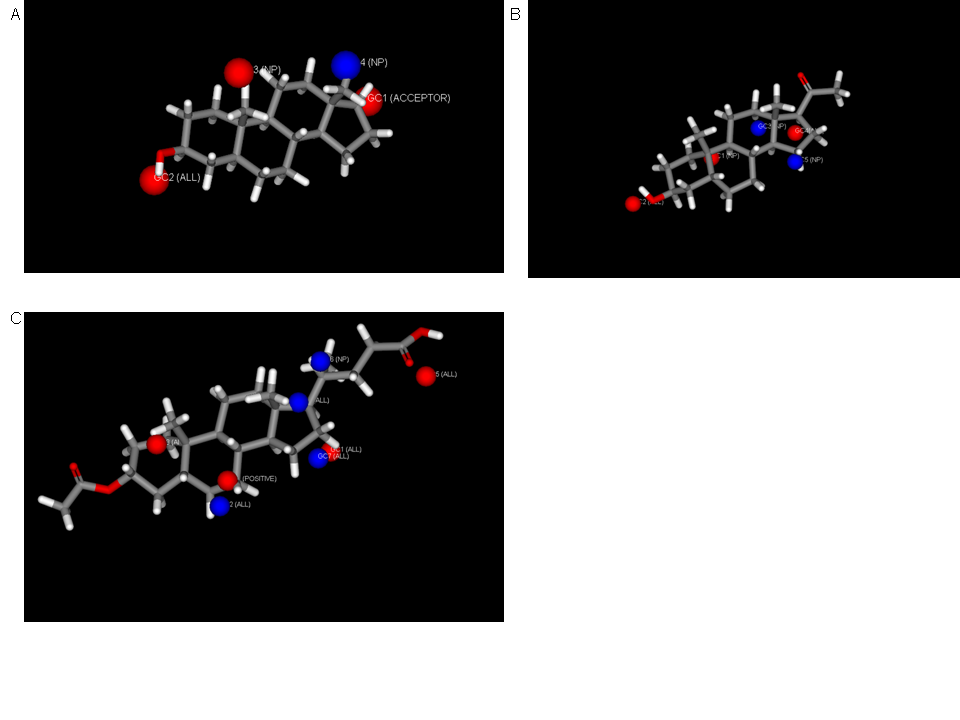

Supplement: Figure S9 — A. 4D-QSAR for androstanes showing the active conformation of 5α-Androstan-3β-ol, B 4D-QSAR for pregnanes showing the active conformation of pregnanolone, C 4D-QSAR for bile acids/salts showing the active conformation of lithocholic acid acetate. (0.19 MB TIF) [file pcbi.1000594.s021.tif]

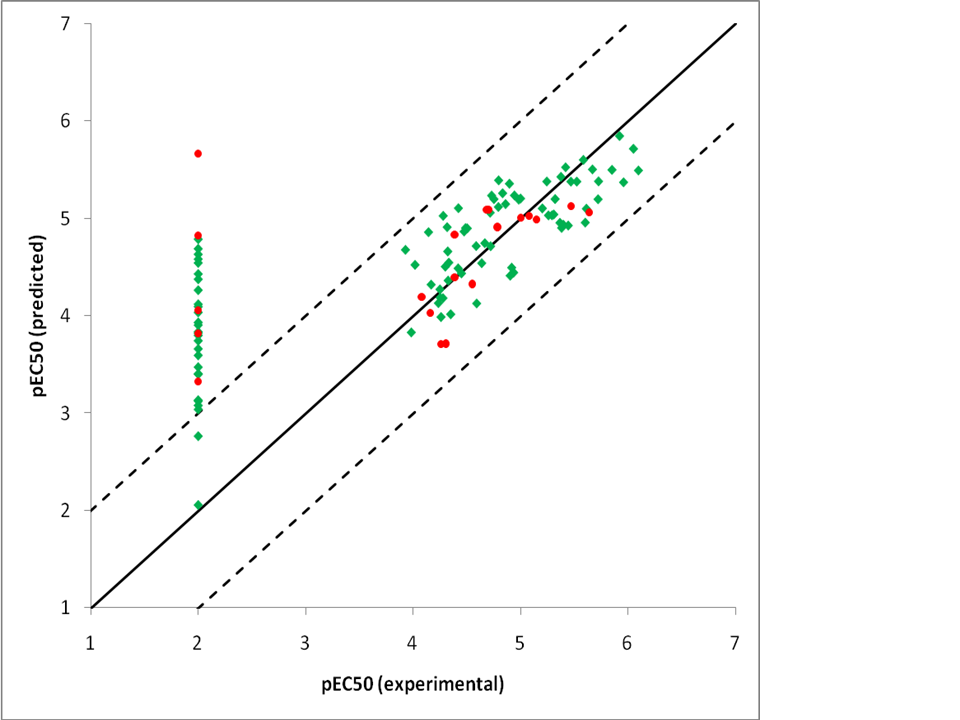

Supplement: Figure S10 — 5D-QSAR Experimental versus predicted pEC50 values for 115 compounds binding to PXR. Training set compounds are displayed in green, test set compounds in red. Threshold compounds are placed at an experimental pEC50 value of 2 to better separate them visually from the other molecules. (0.11 MB TIF) [file pcbi.1000594.s022.tif]
